# Artesunate-loaded thermosensitive chitosan hydrogel promotes osteogenesis of maxillary tooth extraction through regulating T lymphocytes in type 2 diabetic rats

**DOI:** 10.1186/s12903-024-04127-7

**Published:** 2024-03-20

**Authors:** Jinghong Luo, Chen Liang, Kun Chen, Kai Zeng, Rui Bai, Chan Tang, Jiaquan Li, Xiaolin Nong

**Affiliations:** 1 College & Hospital of Stomatology, Guangxi Medical University, No.10 Shuangyong Road, Nanning, Guangxi 530021 China; 2 Guangxi Key Laboratory of Oral and Maxillofacial Rehabilitation and Reconstruction, Nanning, Guangxi 530021 China; 3 Medical Science Research Center, Guangxi Medical University, Nanning, Guangxi 530021 China; 4 Life Science Institute, Guangxi Medical University, Nanning, Guangxi 530021 China

**Keywords:** Artesunate, Hydrogel, Type 2 diabetes mellitus, Osteogenesis, Mitogen-activated protein kinase kinases

## Abstract

**Background:**

Type 2 diabetes mellitus (T2DM) causes severe bone loss after tooth extraction as a hyperglycemic environment causes aberrant bone homeostasis. Artesunate (ART) is known to possess anti-inflammation and osteogenic properties. However, its osteogenesis property in alveolar bone remains unclear. This study aimed to explore the osteogenic and immunoregulatory effects of artesunate-loaded thermosensitive chitosan hydrogel (ART-loaded TCH) on maxilla tooth extraction in T2DM rats.

**Methods:**

T2DM rats were induced by a high-fat diet and streptozotocin. Different concentrations of ART-loaded TCH were applied in tooth extraction sockets. Bone loss and the expression of osteogenic regulatory factors (OPG, ALP, RANK) were evaluated. The immunoregulatory effects of ART-loaded TCH were observed through detecting the infiltration of T lymphocytes and their cytokines. The underlying mechanisms were explored.

**Results:**

Results showed that the 150 mg/ml ART-loaded TCH group significantly ameliorated maxilla bone height and bone mineral density when compared with the T2DM group (*p* < 0.05). It also improved the expression of OPG, ALP, and RANK. Although the alteration of CD4^+^ T, CD8^+^ T, and CD4^+^:CD8^+^ T ratio has no significant difference among groups, the release of Th1 and Th2 in the 150 mg/ml ART-loaded TCH group has been significantly regulated than in the T2DM group (*p* < 0.05). Besides, ART-loaded TCH treatment inhibited the expression of p38 MAPK and ERK1 in T2DM maxilla.

**Conclusions:**

Therefore, the results indicated that 150 mg/ml ART-loaded TCH could be an effective method to prevent bone loss in T2DM tooth extraction rats by modulating the immunoregulation of Th1 and Th2 and the MAPK signaling pathway.

**Supplementary Information:**

The online version contains supplementary material available at 10.1186/s12903-024-04127-7.

## Introduction

The tooth extraction socket initiates a local inflammatory response to prevent the invasion of external pathogens and achieve wound healing [1]. The immune system plays a vital role during the process of bone resorption and remodeling in alveolar bone, as evidenced by the requirement of T lymphocytes in orthodontic tooth movement [2]. It has been found that activated T lymphocytes trigger osteoclastogenesis and damage bone metabolism through mediating pro-osteoclastic cytokines in arthritis and diabetes [3, 4]. Mendiola et al. clarify that activated CD4^+^ T lymphocytes in bone marrow exacerbate insulin resistance and bone loss in type 2 diabetes mellitus (T2DM), which amplifies the importance of T lymphocytes in bone metabolism [4]. Nevertheless, there is limited research that has illustrated the local immunoregulatory function of T lymphocytes in tooth extraction. Cervical lymph nodes are believed to be the gathering place of immune cells in which T lymphocytes complete communication with other immune cells and the process of maturation and differentiation [5]. Thus, it is interesting to explore the regulatory effects of T lymphocytes and their subsets in cervical lymph nodes on alveolar bone metabolism.

The incidence of T2DM in diabetes is up to 90% [6]. T2DM, characterized by hyperglycemia and obesity, is a chronic metabolism disease due to insufficient insulin production or insulin resistance in the pancreas [6, 7]. T lymphocytes mediate the pathological process of T2DM via secreting cytokines, which leads to systemic low-grade inflammation [8]. Long-term chronic inflammatory micro-environment increases the risk of infectious complications in diabetics [7]. Patients with uncontrolled diabetes are highly susceptible to delayed wound healing and disadvantageous post-extraction socket reconstruction [9]. In addition, T2DM exhibits a pronounced alveolar inflammation influenced by a high glucose environment, which impairs collagen metabolism, contributing to hindering bone mineralization and bone turnover [10]. Although T lymphocytes, mediating the pathogenesis of T2DM, are closely associated with bone homeostasis [2], few studies have illustrated the regulatory effects of T lymphocytes in the bone loss of T2DM [4]. This study aimed to explore the relationship between T lymphocyte subsets and osteogenesis in T2DM after tooth extraction.

Artesunate (ART), as a semisynthetic derivation of artemisinin, possesses anti-inflammation and immunoregulatory properties through modulating the T lymphocytes and related cytokines [11]. In recent years, accumulating evidence demonstrated that ART improves bone loss in inflammatory diseases through inhibiting inflammatory cytokines and osteoclast formation [12, 13]. Given that ART has poor water solubility and low oral bioavailability, a suitable drug delivery system is needed to improve its pharmacological properties [14, 15]. Thermosensitive chitosan hydrogel (TCH) has merits in bone regeneration and tissue engineering [16], which also can incorporate ART to enhance its activity and physical stability [17]. Taken together, this study aims to explore the application of ART-loaded TCH on osteogenesis and local T lymphocytes on tooth extraction of T2DM and its potential mechanisms.

## Materials and methods

### Animals and study design

**Fig. 1:**
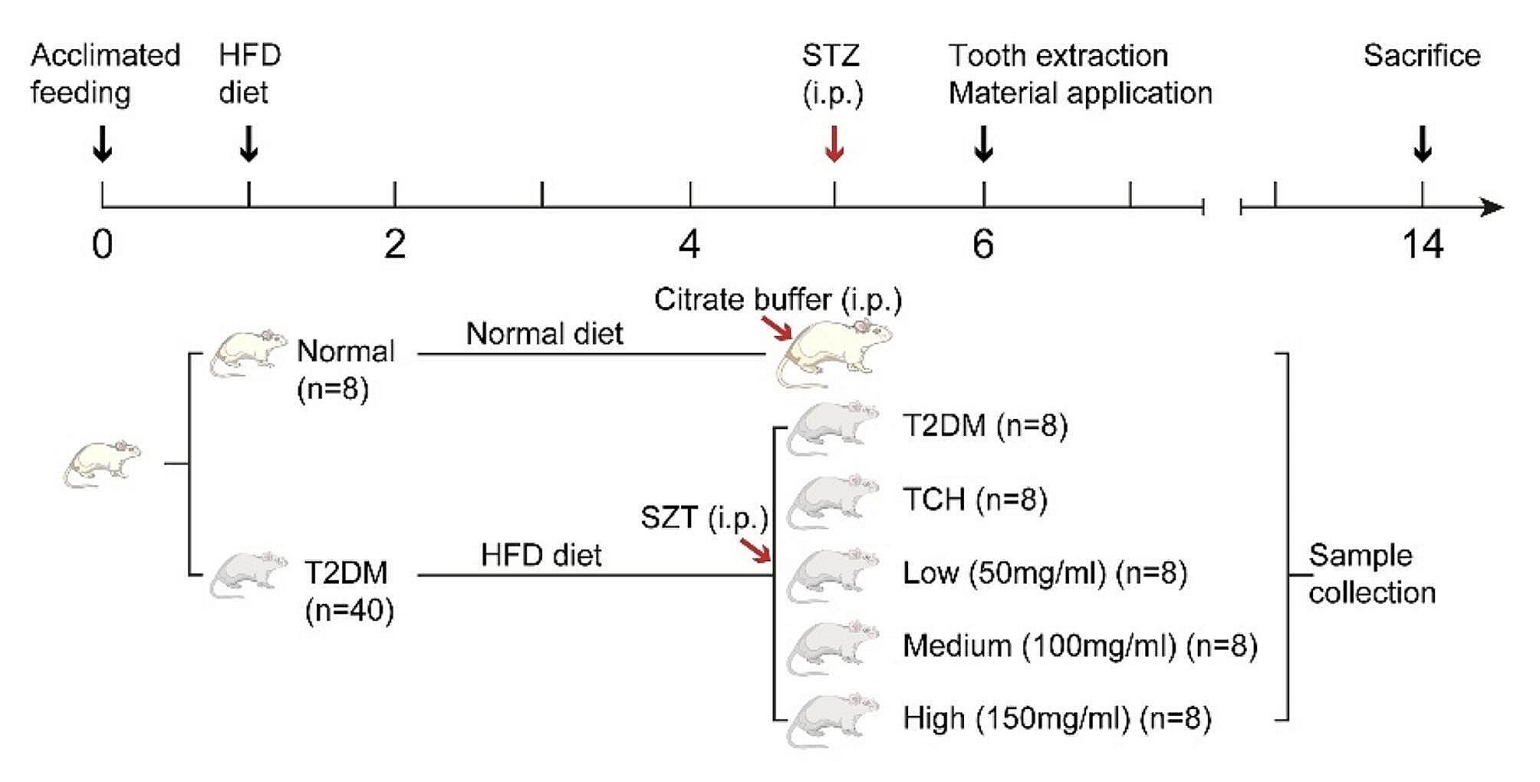
Experimental protocol. T2DM rats were intraperitoneally injected by STZ at 4 weeks after HFD. Tooth extraction surgery was performed at 5 weeks after HFD. Different concentrations of ART-loaded TCH were immediately injected into the socket after tooth extraction in a single injection. Finally, rats were sacrificed at 2 months post tooth extraction. T2DM, type 2 diabetes mellitus group; TCH, thermosensitive chitosan hydrogel group; Low, 50 mg/ml ART-loaded TCH group; Medium, 100 mg/ml ART-loaded TCH group; High; 150 mg/ml ART-loaded TCH group

The animal protocol for this study was in accordance with the Laboratory Animal Guideline for Ethical Review of Animal Welfare and approved by the Animal Experimental Ethics Committee of Guangxi Medical University (GXMU) (No. 202,210,010). This study is in accordance with ARRIVE guidelines. Male Sprague-Dawley rats (weight 120 ± 20 g) were acquired from the Animal Experiment Center of GXMU. Forty-eight rats were used in this study and housed in individually ventilated cages with a specific pathogen-free controlled environment in the Animal Experiment Center of GXMU. The experimental protocol is illustrated (Fig. 1).

### Induction of T2DM

After one week of acclimation, eight rats were randomly selected as the normal group using a random number table and fed on a standard chow diet. The other rats were induced into T2DM rats with a high fat and sugar diet (HFD, boaigang-B1135DM) and a low dose of streptozotocin (STZ). All rats were fasted for 12 h after 4 weeks of the HFD diet. The HFD rats were injected intraperitoneally with a single dose of STZ (40 mg/kg), while the normal rats were administrated with the same volume of citrate buffer according to previous studies [18]. Fasting blood glucose (FBG) was measured on the 3rd, 7th, 30th, and 60th days after STZ administration using Accu-Chek Active test strips. The rats with FBG concentration > 11.1mmol/L on the 7th day after STZ were considered T2DM rats. Certain rats that had not been successfully established as T2DM rats at the first time would be injected with the same amount of STZ again after their blood sugar had completely returned to normal.

### Preparation of artesunate-loaded thermostatic chitosan hydrogel (ART-loaded TCH)

Different amounts of artesunate (50, 100,150 mg) (Guilin Pharmaceutical Co., Ltd., Guilin) were dissolving in the lipid phase of thermostatic chitosan hydrogels (TCH, Huizhong International Medical Devices Co., Ltd, Beijing) based on previous studies [14, 17].

### Tooth extraction

The T2DM rats were successfully induced and then subjected to tooth extraction. The rats were anesthetized intraperitoneally and locally using 2% pentobarbital sodium (50 mg/kg) and articaine respectively. Afterward, the right maxillary first and second molars were extracted using small forceps [19], and then the materials were applied to the tooth extraction sockets immediately according to the following groups, T2DM, TCH, 50 mg/ml ART-loaded TCH, 100 mg/ml ART-loaded TCH, 150 mg/ml ART-loaded TCH.

### Intraperitoneal glucose tolerance test (IPGTT)

An Intraperitoneal Glucose Tolerance Test (IPGTT) was conducted to test the glucose tolerance in the seventh week of the experiment. The 12-h-fasted rats received an intraperitoneal injection of glucose solution (2 g/kg) and their blood glucose levels were tested at 0, 30, 60, 90, and 120 min post-injection. The area under the curve (AUC) and homeostatic model assessment of insulin resistance (HOMA-IR) were calculated [18, 20]. The equation of HOMA-IR was FBG (mmol/L) ×FINS (mU/mL)/22.5. Fasting serum insulin level was determined by ELISA kits (Jiubang Biotech Co Ltd. Quanzhou, China).

### Flow cytometry

Peripheral blood mononuclear cells and lymphocytes were isolated and washed using Ficoll density gradient centrifugation and grinding methods, respectively. Resuspended cells were first treated with FcR-block. Two panels of monoclonal antibodies cocktail were set to examine the subsets of T lymphocytes (Additional file 1). Initially, a cell stimulation cocktail (Invitrogen, USA) was added to the cells and then incubated in a CO_2_ incubator for 4 h. The cells were stained with surface antibodies and fixable viability dye (eBioscience, USA) before being treated with fixation and permeabilization buffer. Finally, intracellular staining was performed to detect the release of cytokines. The cells were resuspended and analyzed by flow cytometry (BD FACSCanto™, USA). Fluorescence minus one test and isotype controls were used for compensation of fluorescence. A minimum of 30,000 events in main cells were acquired for each specimen in lymphocytes and 10,000 events in blood samples. The acquired data were analyzed by FlowJoV10.8.

### Micro-computed tomography (Micro-CT)

Micro-CT was performed on day 0 and day 60 after tooth extraction. Rats under anesthesia of 2% pentobarbital sodium (50 mg/kg) were fixed in the scan tube of LCT-200 Micro-CT (Aloka Corporation, Japan). The maxilla were scanned with voxel size 80*80 µm and slice thickness 80 μm. Maxilla bone height of the second molar at buccal, middle, and palatal and its bone mineral density (BMD) were measured in Mimics Research (V 21.0, Materialise, Belgium) to compare the bone loss in each group.

### Histological analysis

All samples including the maxilla and lymph node were firstly fixed in 4% paraformaldehyde for 24 h. Maxilla were then decalcified in 10% ethylene diamine tetraacetic acid for 3 months before being embedded in paraffin. Serial Sect. (3 μm) of maxilla samples were cut from the mesial of the second molar to the distal. Deparaffinized sections were stained with hematoxylin and eosin and immunohistochemistry staining (MXB Biotechnologies) according to the manufacturer’s protocol. Primary antibodies used were GATA-3 (1:50, proteintech), IFN-γ (1:2000, Bioss), IL-4 (1:200, proteintech), OPG (1:500, proteintech), ALP (1:2000, proteintech), RANK (1:2000, Bioss), ERK1 (1:500, Bioss), and p38 MAPK (1:50, Bioss). Negative controls were used to test immunostaining specificity by replacing the primary antibody with phosphate buffer saline.

### Real-time quantitative PCR (RT-qPCR) analysis

Total RNA from the maxilla and lymph node were extracted using TRIzol reagent (Invitrogen, USA). RT-qPCR was performed using ChamQ Universal SYBR qPCR Master Mix (Vazyme) according to the manufacturer’s instructions. Endogenous housekeeping gene β-actin was used. The experimental data were quantified using the 2^−ΔΔCt^ method. Each sample was triplicated independently. The primer sequences used are listed in Additional file 2.

### Western blot analysis

The lymph nodes and maxilla were lysed by RIPA (Invitrogen) with phosphatase and protease inhibitors to extract protein as stated by the manufacturer’s protocol. The concentration of total protein was measured and then subjected to electrophoresis in SDS-PAGE gel. Protein in the gel was transferred to PVDF membranes through the semi-dry electrophoretic transfer unit. Afterward, skimmed milk powder was used to block the non-specific binding sites. The membranes were then incubated overnight with primary antibodies including rabbit anti-β-actin (1:20000, Bioss), mouse anti-GATA-3 (1:2000, Proteintech), mouse anti-IL-4 (1:1000, Proteintech), rabbit anti-T-BET (1:500, Proteintech), rabbit anti-phospho-p38 MAPK (1:1000, Bioss), rabbit anti-p38 MAPK (1:1000, Bioss), mouse anti-ERK1 (1:1000, Bioss), mouse anti-phospho-ERK1 (1:1000, Bioss). Finally, secondary antibodies were incubated with anti-rabbit (1:10000, Bioss) or anti-mouse (1:10000, Bioss) before detecting the HRP signal using ECL.

### Network pharmacology analysis

On November 20th, 2023, the disease-related genes of T2DM were downloaded from databases including TTD, DrugBank, DisGeNET, KEGG, PHARMGKB, and GeneCards. “Type 2 diabetes mellitus” was used as the keyword to obtain regulatory genes. ART-targeted genes were obtained from DrugBank, STITCH, and the Swiss Target Prediction database. All obtained data were screened and normalized. ART-targeted pathways in T2DM were analyzed by the Enriched Kyoto Encyclopedia of Genes and Genomes (KEGG) (*p* < 0.05, FDR < 0.05).

### Molecular docking of artesunate to targeted protein

PubChem and the UniProt database were used to download the structure of artesunate (Compound CID: 6,917,864) and the three-dimensional structure of target proteins (ERK1: P21708, p38 MAPK: P70618), respectively. The protein structures were removed solvent molecules and ligands, deleted water, and added all hydrogens through AutoDock software. Molecular docking energy was calculated by the Lamarckian Genetic Algorithm. The binding energy < -5.0 kJ/mol was considered the optimal conformational binding structure [21].

### Statistical analysis

Data analyzed by SPSS are illustrated as mean ± standard deviation. Each experiment was conducted three times independently. Statistical significance was determined among groups by using a one-way analysis of variance if the data met the standard of tests of normality and homogeneity of variances, otherwise using the Kruskal-Wallis test. The data normality and homogeneity of variances were tested by Shapiro-Wilk and Levene Statistics respectively. Statistically different is designated as *p < 0.05 and **p < 0.01.

## Results

### T2DM tooth extraction rats were successfully induced

**Fig. 2:**
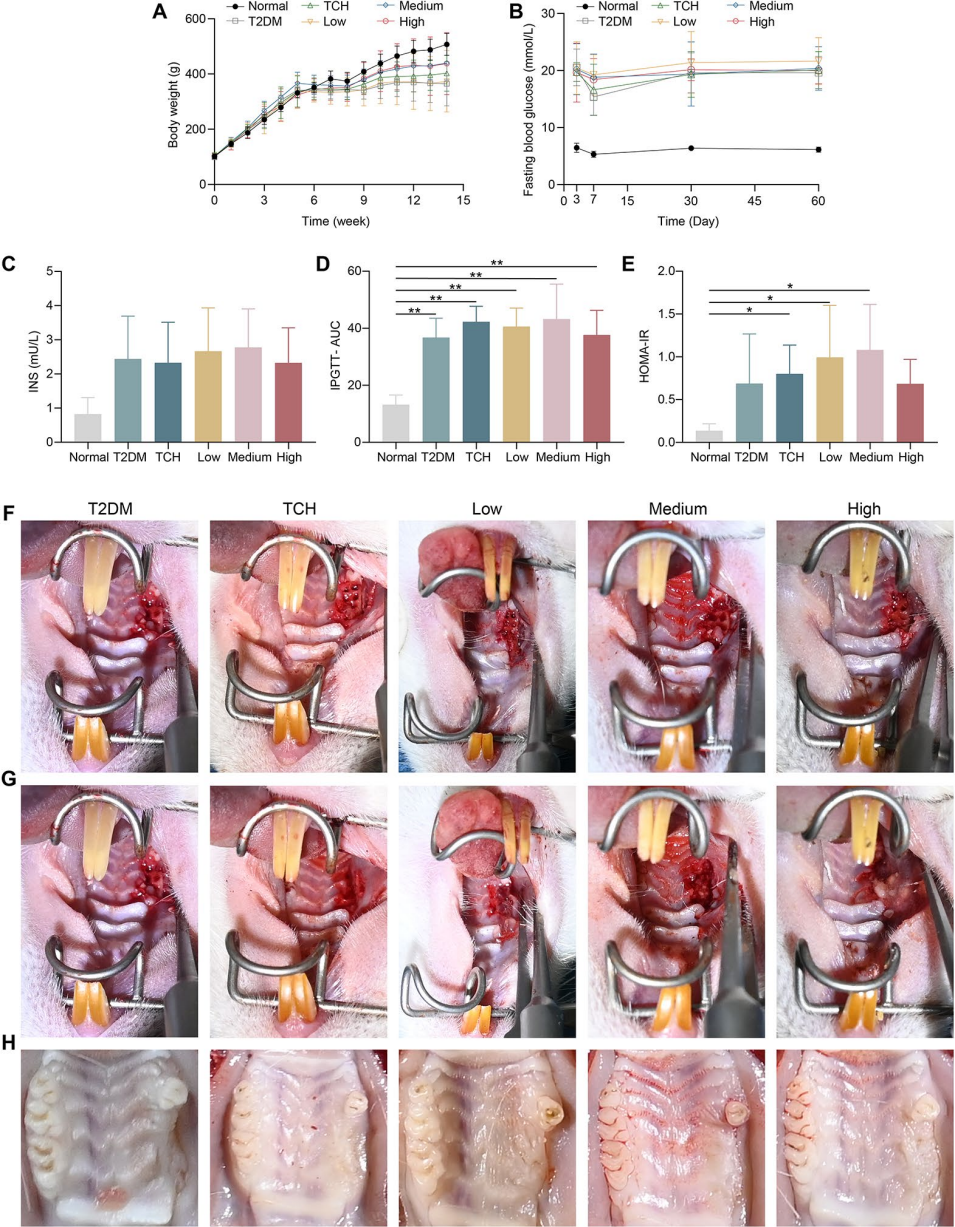
T2DM tooth extraction rats were successfully established. **A–C** Body weight, fasting blood glucose, and fasting insulin levels of each group (8 rats per group). **D, E** Intraperitoneal glucose tolerance test (IPGTT) was performed at week 7 (*n* = 8). The AUC of T2DM rats significantly increased in T2DM, TCH, and 50 mg/ml, 100 mg/ml, and 150 mg/ml ART-loaded TCH groups than in normal rats (*p* < 0.01) (**D**). The HOMA-IR of T2DM rats significantly increased in TCH, 50 mg/ml, and 100 mg/ml ART-loaded TCH groups than in normal rats (*p* < 0.05) (**E**). **F–H** The first and second molars of the maxilla in T2DM rats were extracted (**F**) and immediately applied different concentrations of ART-loaded TCH (**G**). The healing of the socket was recorded at 2 months after tooth extraction (**H**). Data were presented as mean ± standard deviation. *p < 0.05 and **p < 0.01. T2DM, type 2 diabetes mellitus group; TCH, thermosensitive chitosan hydrogel group; Low, 50 mg/ml ART-loaded TCH group; Medium, 100 mg/ml ART-loaded TCH group; High; 150 mg/ml ART-loaded TCH group

During the experiment, T2DM rats exhibited body weight loss after injecting with STZ when compared with the normal rats. Compared with the T2DM group, the 100 mg/ml and 150 mg/ml ART-loaded TCH groups improved body weight (Fig. 2A). Besides, the levels of FBG in T2DM rats exceeded 11.1 mmol/L on the 7th day after STZ injection (Fig. 2B). The fasting insulin levels increased in T2DM rats than in normal rats (Fig. 2C), however, it has been determined as not statistically significant (*p* > 0.05). The glucose tolerance in T2DM rats was more seriously impaired than in normal rats evidenced by the AUCand the HOMA-IR value significantly increasing (*p* < 0.05) (Fig. 2D-E). Ultimately, the first and second molars of the maxilla in T2DM rats were extracted, and immediately applied different concentrations of ART-loaded TCH (Fig. 2F-G). The healing of the socket was recorded at 2 months after tooth extraction (Fig. 2H).

### Effects of ART-loaded TCH on osteogenesis in tooth extraction socket in T2DM rats

**Fig. 3:**
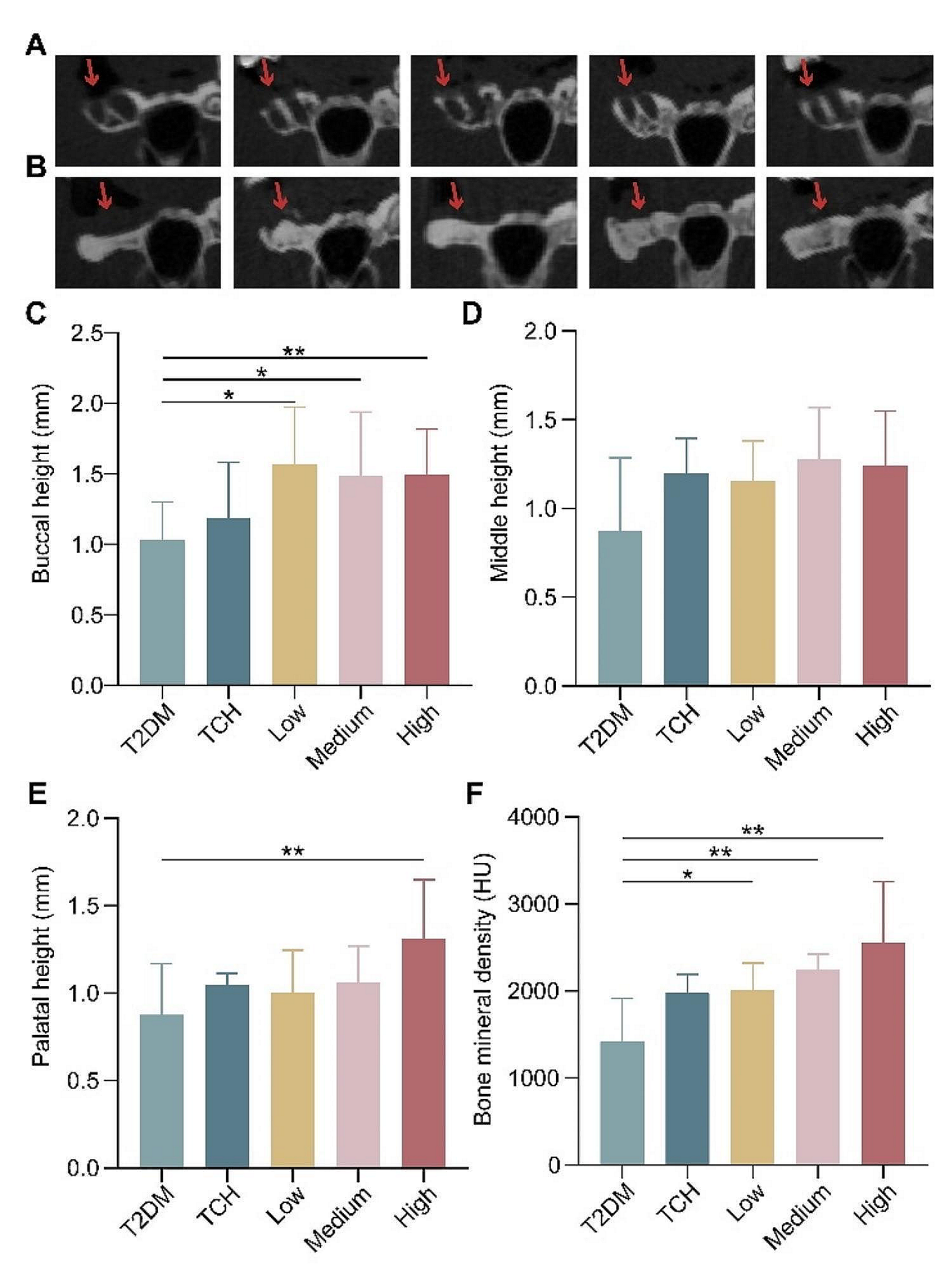
ART-loaded TCH alleviated maxilla bone loss on tooth extraction sockets of T2DM. **A, B** Representative images of the maxilla by micro-CT at day 0 (**A**) and day 60 (**B**) after tooth extraction in each group. **C–E** The loss of maxilla bone height of buccal (**C**), middle (**D**), and palatal (**E**) after tooth extraction in T2DM rats (n ≧ 3). **F** Bone mineral density of the maxilla after extracting the tooth in T2DM rats (n ≧ 3). Compared with the T2DM group, the 150 mg/ml ART-loaded TCH group significantly alleviated maxilla bone height and Bone mineral density (*p* < 0.01). *p < 0.05 and **p < 0.01. T2DM, type 2 diabetes mellitus group; TCH, thermosensitive chitosan hydrogel group; Low, 50 mg/ml ART-loaded TCH group; Medium, 100 mg/ml ART-loaded TCH group; High; 150 mg/ml ART-loaded TCH group

To evaluate the osteogenesis of ART-loaded TCH on tooth extraction sockets in T2DM rats, the maxilla bone height and bone mineral density were estimated by micro-CT (Fig. 3A-B). The buccal, middle, and palatal bone height of the maxilla and bone mineral density in T2DM rats decreased at 2 months after tooth extraction (Fig. 3C-F). Compared with the T2DM group, the 150 mg/ml ART-loaded TCH group significantly ameliorated the buccal and palatal bone height of the maxilla and bone mineral density (*p* < 0.01). In addition, the 50 mg/ml and 100 mg/ml ART-loaded TCH groups significantly ameliorated the buccal bone height of the maxilla and bone mineral density than the T2DM group (*p* < 0.05). However, there is no significant difference among TCH, 50 mg/ml, 100 mg/ml, and 150 mg/ml ART-loaded TCH.

**Fig. 4:**
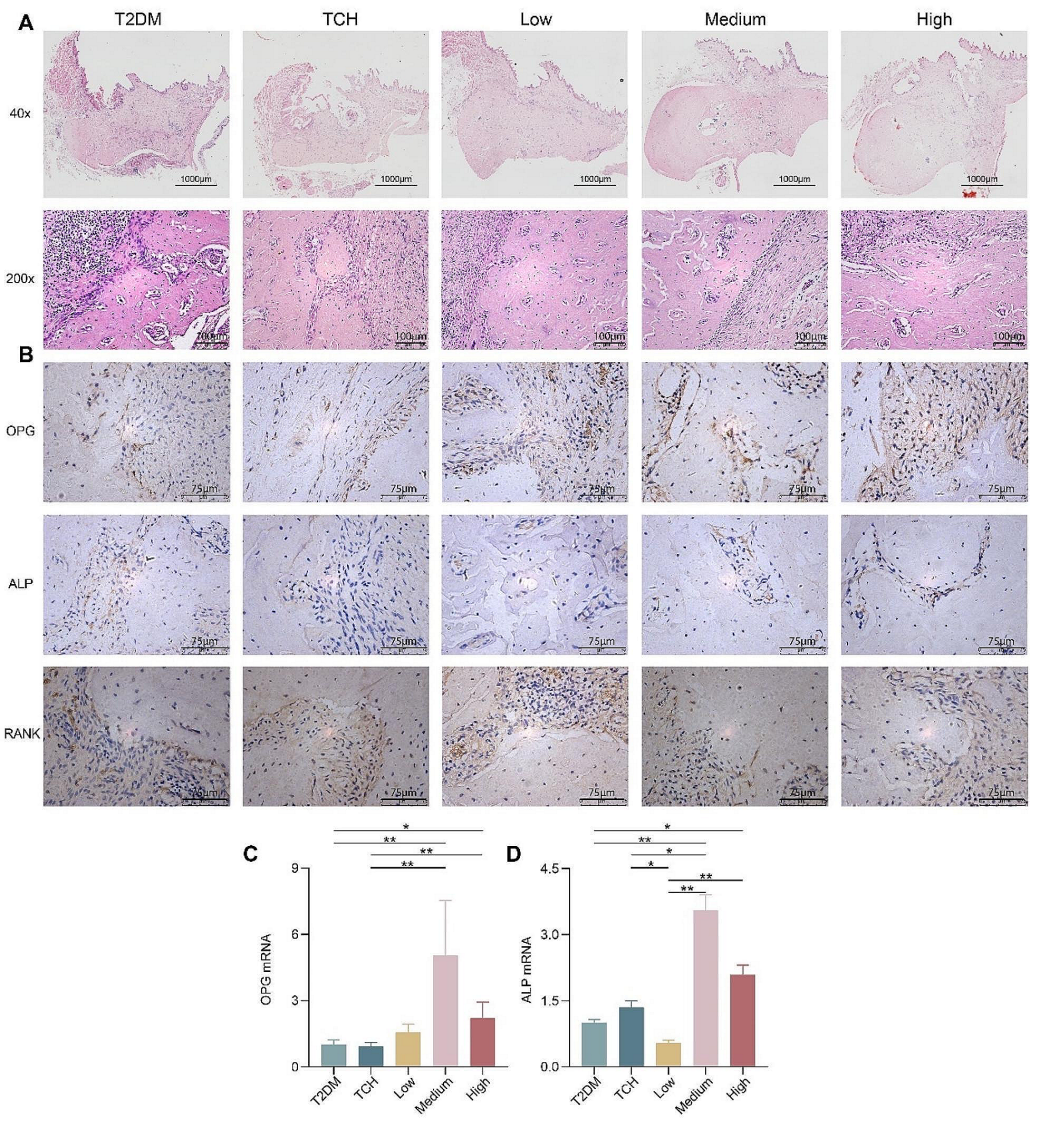
ART-loaded TCH alleviated osteogenic regulatory factors on tooth extraction sockets of T2DM. **A** The histology of the maxilla was observed by H&E. **B–D** The expression of OPG (**B, C**), ALP (**B, D**), and RANK (**B**) in maxilla tooth extraction sockets was detected by IHC and RT-qPCR (*n* = 3). The expression of OPG and ALP greatly improved in 100 mg/ml and 150 mg/ml ART-loaded TCH groups than in the T2DM group (*p* < 0.05) (**C, D**). Data are shown as mean ± standard deviation. *p < 0.05 and **p < 0.01. T2DM, type 2 diabetes mellitus group; TCH, thermosensitive chitosan hydrogel group; Low, 50 mg/ml ART-loaded TCH group; Medium, 100 mg/ml ART-loaded TCH group; High; 150 mg/ml ART-loaded TCH group

Furthermore, the morphology and histology of the maxilla were observed by H&E. ART-loaded TCH ameliorated the inflammation infiltration in the maxilla of T2DM rats accompanied by less bone loss (Fig. 4A). To explore the osteogenic effects of ART-loaded TCH, the expression of OPG, ALP, and RANK was detected. Results indicated that the expression of OPG and ALP in maxilla tooth extraction sites was significantly upregulated in the 100 mg/ml and 150 mg/ml ART-loaded TCH groups when compared with the T2DM group (*p* < 0.05), whereas the expression of RANK showed the opposite trend (Fig. 4B-D).

### Effects of ART-loaded TCH on regulating CD4^+^ and CD8^+^T in cervical lymph nodes and peripheral blood

**Fig. 5:**
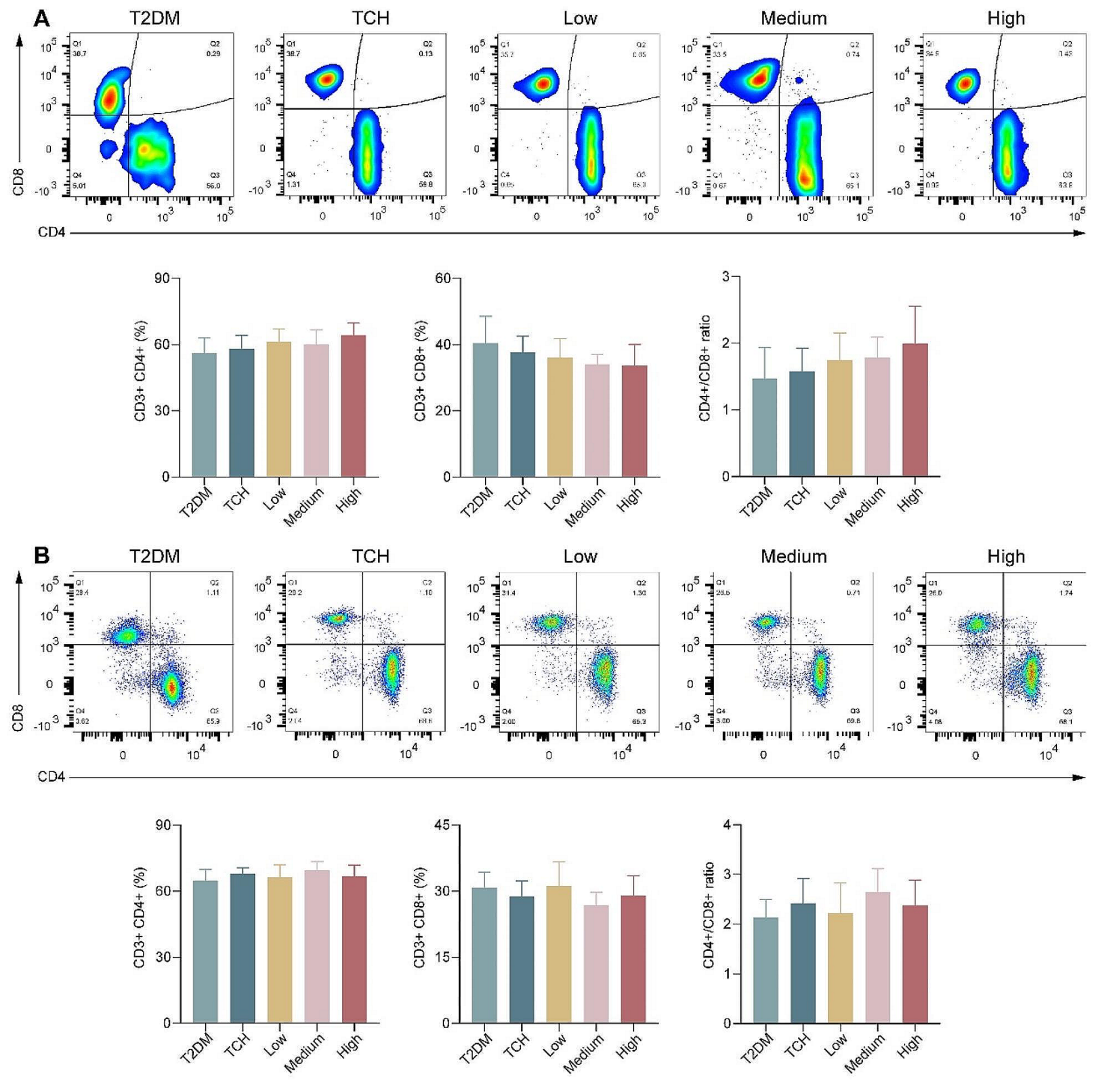
ART-loaded TCH mildly mediated the percentage of CD4^+^ and CD8^+^ T lymphocytes in T2DM rats. **A, B** The change of CD4^+^, CD8^+^, and CD4^+^:CD8^+^ T lymphocytes in peripheral blood (**A**) and cervical lymph nodes (**B**) in each group (*n* ≥ 6). Data are shown as mean ± standard deviation. *p < 0.05 and **p < 0.01. T2DM, type 2 diabetes mellitus group; TCH, thermosensitive chitosan hydrogel group; Low, 50 mg/ml ART-loaded TCH group; Medium, 100 mg/ml ART-loaded TCH group; High; 150 mg/ml ART-loaded TCH group

The proportion of immune cells in cervical lymph nodes can reflect the local inflammatory infiltration in the maxilla of T2DM and the proportion of immune cells in peripheral blood can reflect the systemic inflammatory infiltration. To investigate the gathering and infiltration of T lymphocytes in peripheral blood (Fig. 5A) and cervical lymph nodes (Fig. 5B), flow cytometry was performed to detect the percentage of CD4^+^ and CD8^+^ T lymphocytes. The results demonstrated that the percentage of CD4^+^ T lymphocytes and the ratio of CD4^+^:CD8^+^ T increased accompanied by the increasing concentration of ART-loaded TCH, while the percentage of CD8^+^ T lymphocytes decreased. However, there is no significant difference between the treatment groups and the T2DM group (*p* > 0.05).

### Effects of ART-loaded TCH on regulating Th1, Th2, and inflammatory factors in cervical lymph nodes

**Fig. 6:**
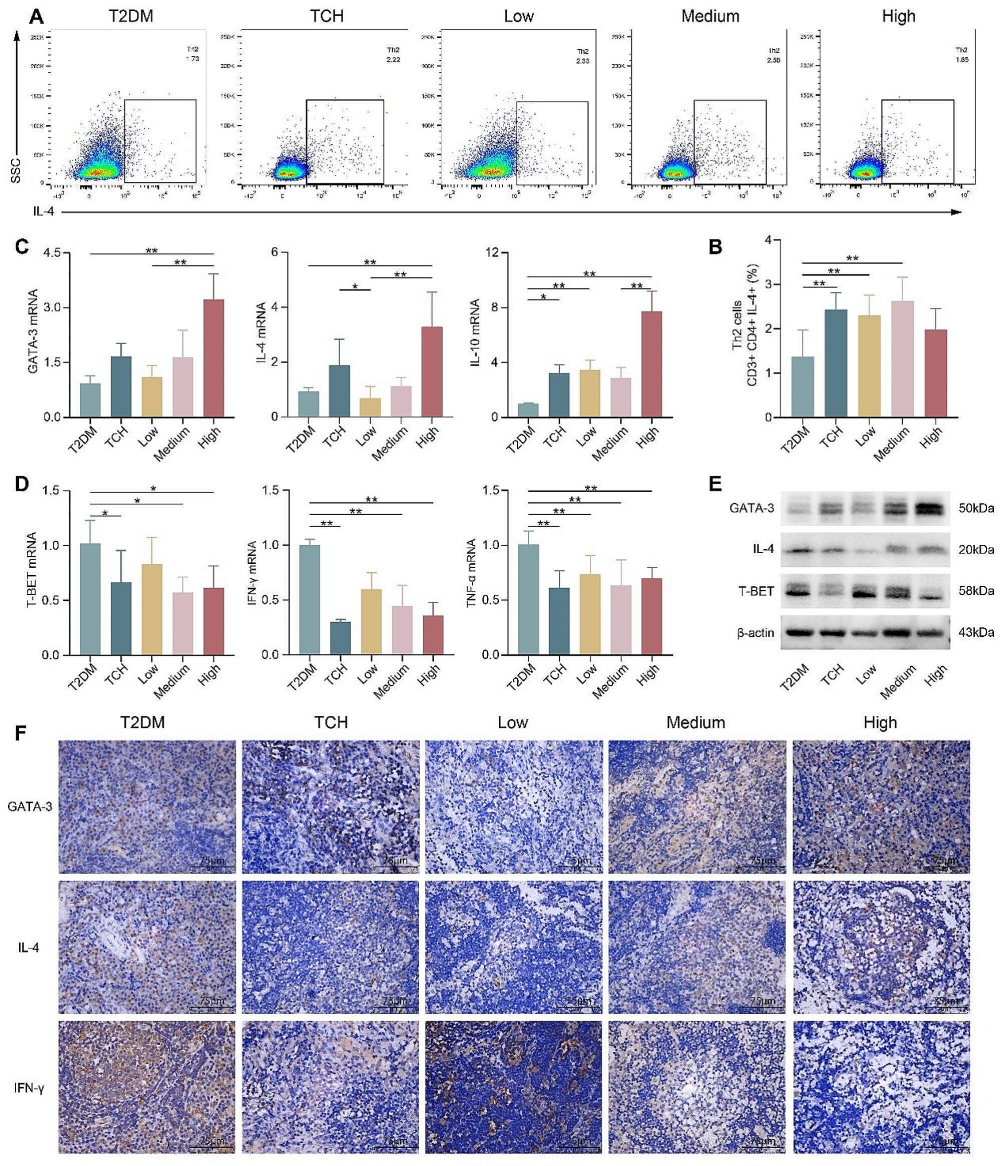
ART-loaded TCH ameliorated the infiltration of Th1, Th2, and inflammatory cytokines in cervical lymph nodes. **A-B** Flow cytometry was used to detect the percentage of Th2 (*n* ≥ 6). The TCH and 50 mg/ml and 100 mg/ml ART-loaded TCH groups significantly increased the percentage of Th2 than the T2DM group(*p* < 0.01). **C, D** PCR was used to estimate the mRNA expression of Th1 (T-BET) and Th2 (GATA-3) and related secreted cytokines, including IL-4, IL-10, IFN-γ, and TNF-α (*n* = 3). The 150 mg/ml ART-loaded TCH group remarkably regulated the infiltration of the above cells and cytokines when compared with the T2DM group (*p* < 0.01). **E, F** The protein expression of GATA-3, IL-4, T-BET, and IFN-γ were detected by WB and IHC (*n* = 3). The uncropped blots are displayed in Additional file 3. Data are shown as mean ± standard deviation. *p < 0.05 and **p < 0.01. T2DM, type 2 diabetes mellitus group; TCH, thermosensitive chitosan hydrogel group; Low, 50 mg/ml ART-loaded TCH group; Medium, 100 mg/ml ART-loaded TCH group; High; 150 mg/ml ART-loaded TCH group

To further investigate the regulatory function of ART-loaded TCH on CD4^+^ T subsets, we detected the infiltration of Th1 and Th2 in lymph nodes through examining their gene and protein expressions. Flow cytometry displayed a profound increase in the percentage of Th2 in the TCH and 50 mg/ml and 100 mg/ml ART-loaded TCH groups (*p* < 0.01) than in the T2DM group (Fig. 6A-B). Results indicated that the expression of GATA-3 (transcription factor of Th2) was significantly upregulated (*p* < 0.01) (Fig. 6C, E-F), while the expression of T-BET (transcription factor of Th1) was significantly downregulated in the 150 mg/ml ART-loaded TCH group when compared with the T2DM group (*p* < 0.05) (Fig. 6D-E). Moreover, the secretion of pro-inflammatory cytokines by Th1 like IFN-γ (Fig. 6D, F) and TNF-α (Fig. 6D) was significantly decreased (*p* < 0.01), while anti-inflammatory cytokines secreted by Th2, like IL-4 (Fig. 6C, E-F) and IL-10 (Fig. 6C) were distinctly increased (*p* < 0.01) in the 150 mg/ml ART-loaded TCH group than in the T2DM group.

### The mechanism of ART-loaded TCH on osteogenesis in the tooth extraction socket in type 2 diabetes rats

**Fig. 7:**
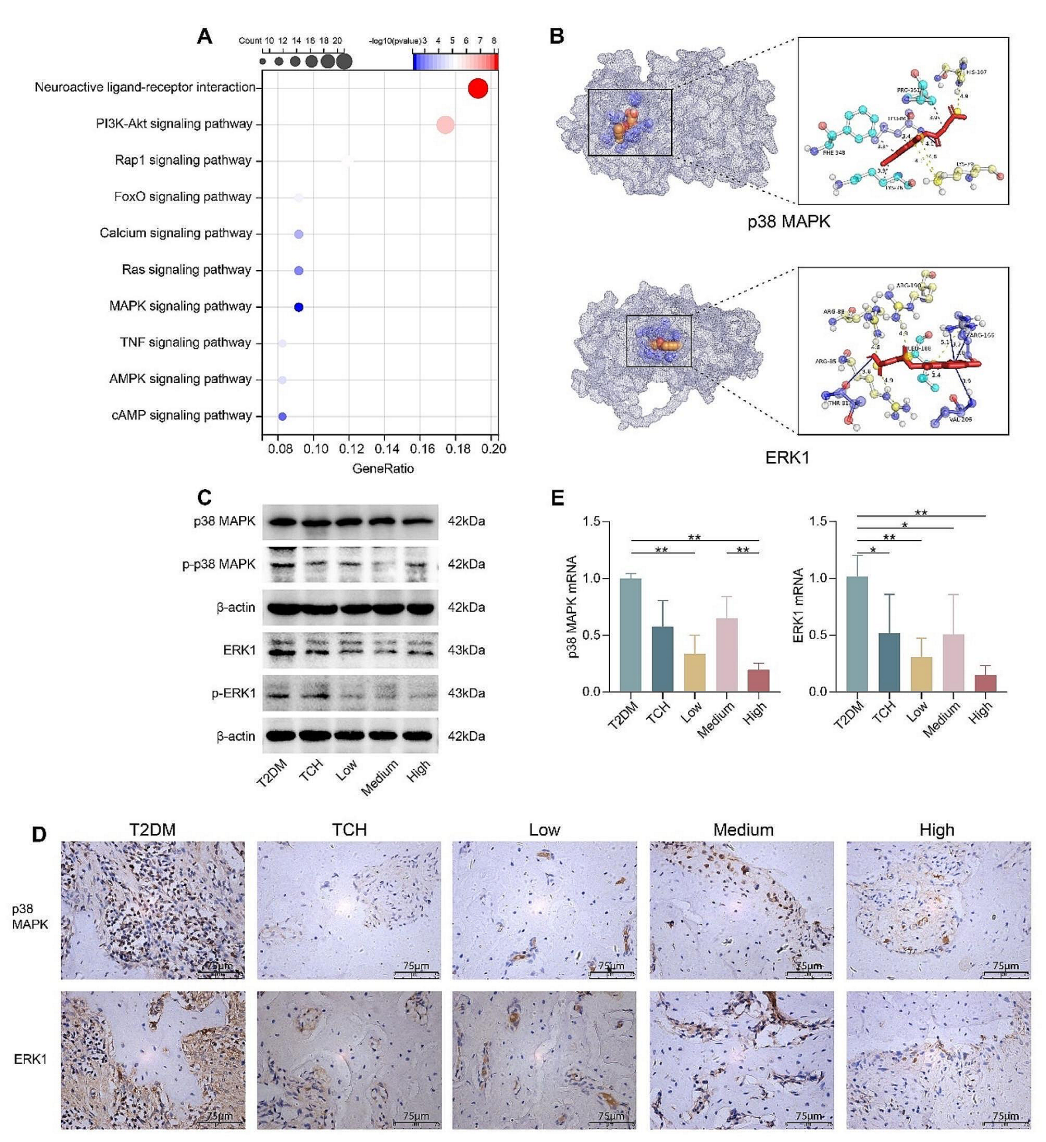
ART-loaded TCH suppressed MAPK signaling pathway in the maxilla of T2DM tooth extraction. **A** Network pharmacology analysis revealed the potential immunoregulatory mechanism of ART in T2DM. The top ten enriched pathways were illustrated. **B** Molecular docking of ART to MAPK and ERK1 proteins. **C, D** The protein levels of p38-MAPK, p-p38-MAPK, ERK1, and p-ERK1, were analyzed by WB and IHC (*n* = 3), which can be downregulated by ART-loaded TCH treating. The uncropped blots are displayed in Additional file 3. **E** Relative mRNA expression of MAPK (**D**) and ERK1 (**E**) was analyzed by PCR (*n* = 3). The 150 mg/ml ART-loaded TCH group significantly decreased the mRNA expression of MAPK and ERK1 than the T2DM group (*p* < 0.01). Data are shown as mean ± standard deviation. *p < 0.05 and **p < 0.01. T2DM, type 2 diabetes mellitus group; TCH, thermosensitive chitosan hydrogel group; Low, 50 mg/ml ART-loaded TCH group; Medium, 100 mg/ml ART-loaded TCH group; High; 150 mg/ml ART-loaded TCH group

To explore the potential mechanisms of ART-loaded TCH on osteogenesis, we conducted network pharmacology analysis and molecular docking. The potential mechanisms of ART on T2DM were investigated by network pharmacology analysis. After excluding the duplicates, 8918 genes related to type 2 diabetes mellitus were gained from TTD, DrugBank, DisGeNET, KEGG, and PHARMGKB databases. In addition, DrugBank, STITCH, and the Swiss Target Prediction database were used to search the potential ART-targeted genes. We found 124 potential targeted genes. The potential genes that can be regulated by ART in T2DM were screened and analyzed by KEGG pathway enrichment. The top ten potential regulatory mechanisms related to signal transduction are listed below (Fig. 7A). Based on the previous studies, we supposed that the MAPK signaling pathway might be the vital mediator of bone loss of T2DM tooth extraction.

Besides, molecular docking predicted that ART could form a stable bond with MAPK signaling markers, including ERK1 and p38-MAPK with a low binding energy (< −5 kcal/mol) (Fig. 7B). The binding energy formed between ART and p38-MAPK is −9.09 kcal/mol, ART and ERK1 is −6.77 kcal/mol. Furthermore, we performed PCR, WB, and IHC to test the effects of ART-loaded TCH on MAPK signaling markers. We validated that the 150 mg/ml ART-loaded TCH group significantly decreased the expression of p38-MAPK and ERK1 and the phosphorylation levels of p38-MAPK and ERK1 when compared with the T2DM group (*p* < 0.01) (Fig. 7C-E).

## Discussion

This study demonstrated that ART-loaded TCH has great therapeutic effects on osteogenesis and immunomodulation in T2DM tooth extraction rats. The T2DM model was successfully established as deduced body weight and raised FBG and insulin resistance. Single administration of different concentrations of ART-loaded TCH has great impacts on maxilla bone height and bone mineral density. The application of ART-loaded TCH not only significantly regulated the expression of osteogenic factors including OPG, ALP, and RANK, but also greatly modulated the infiltration of Th1, Th2, and their related factors, like IFN-γ, TNF-α, IL-4, and IL-10. However, ART-loaded TCH moderately regulated the percentages of CD4^+^ T and CD8^+^ T lymphocytes with no significant difference. Notably, the interaction between osteogenesis and immune cells is closely connected, and the underlying mechanism regulated by ART-loaded TCH is of interest.

T2DM is a prevalent metabolic disease with multiple complications, including slowing wound healing and recurrent infections [6]. T2DM can interfere with and hinder the healing of extraction sockets by inhibiting osteogenesis differentiation of mesenchymal stem cells [22]. Although extensive studies revealed that local medication treatment effectively promoted bone regeneration, few studies have clarified their potential application in diabetic tooth extraction sockets [23, 24]. It might be due to chronic system inflammation in diabetes restricting medicine’s efficacy. ART is effective in inhibiting inflammation and promoting osteogenesis [13]. Considering the stability and solubility profiles of ART, a suitable delivery system, like TCH, could efficiently exert its effects [17]. In the present study, we clarified the therapeutic impact of ART-loaded TCH on osteogenesis and local T lymphocyte response of tooth extraction sockets in T2DM rats.

In the current study, a low-dose streptozotocin-induced T2DM model exhibited typical symptoms including body weight loss, hyperglycemia, decreased insulin, and insulin resistance, consistent with previous studies [4, 18]. We noticed that TCH and ART-loaded TCH treatments at the tooth extraction site have no significant therapeutic effects on ameliorating these typical characteristics. TCH is an excellent local delivery system with non-toxicity, biocompatible, and biodegradability [25]. Physiological temperatures can turn a liquid state at low temperatures into a stable gel state without any side effects [26]. It has been commonly used in bone and tissue regeneration for its persistent drug-controlled release function [27]. Our results showed that TCH improved osteogenesis on tooth extraction sockets in T2DM rats, and 150 mg/ml ART-loaded TCH achieved a better osteogenic effect when compared with other treatment groups.

**Fig. 8:**
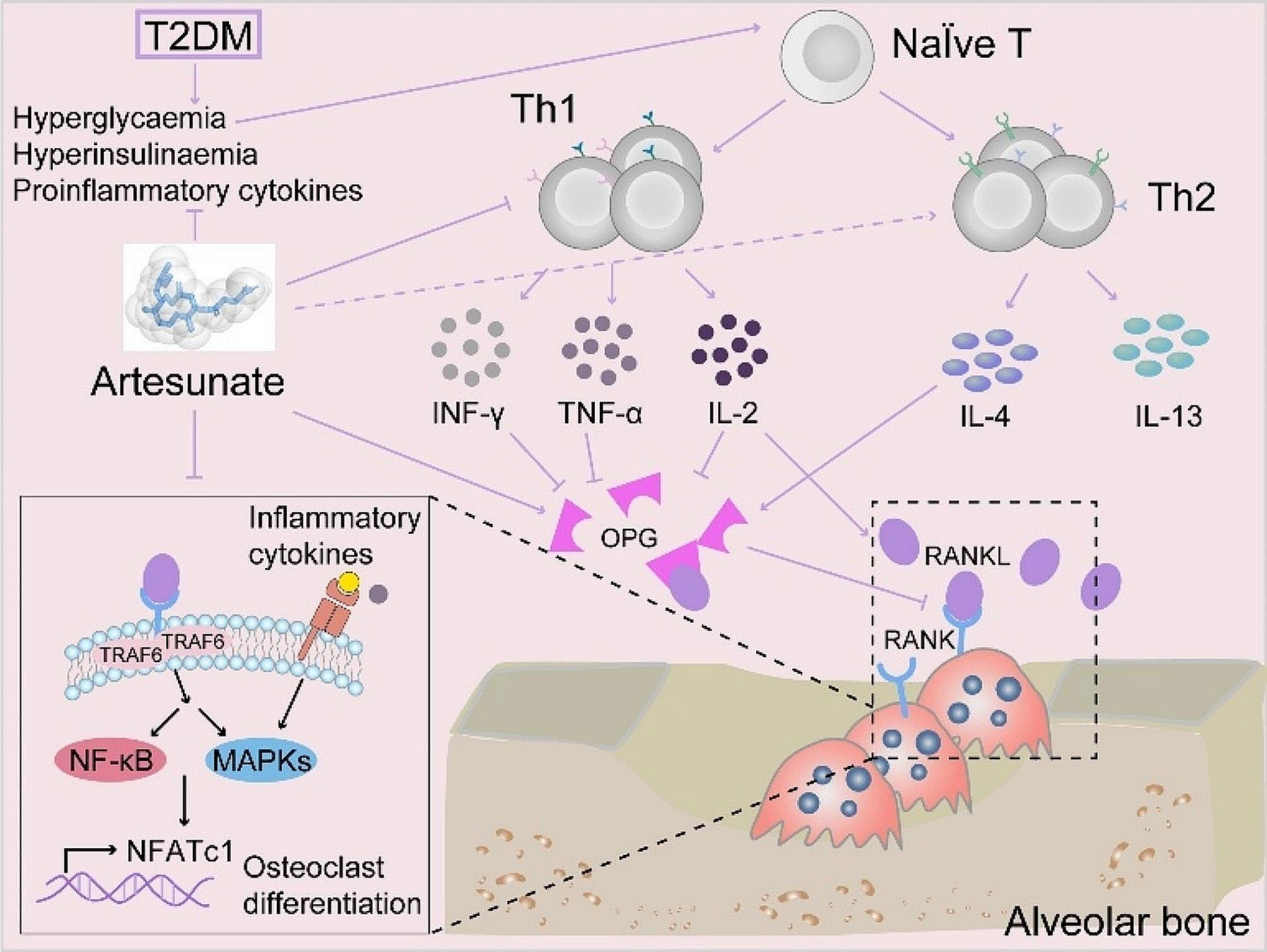
Illustration of the proposed mechanism of ART-loaded TCH promoted osteogenesis and restrains local T lymphocytes immune through suppressing MAPKs pathway in T2DM tooth extraction sockets

ART has been studied extensively and is established for its anti-inflammation and immunoregulation [28, 29]. Recently, accumulating evidence pointed out that ART inhibits osteoclastogenesis and bone loss through suppressing inflammation factors and reactive oxygen species [30, 31]. This study for the first time investigated the local application of ART in tooth extraction sockets. The osteogenic effect of ART-loaded TCH has been confirmed in this study as the bone mineral density, bone height, and the expression of osteoprotegerin (OPG) and alkaline phosphatase (ALP) increased in the ART-loaded TCH treatment group, while the expression of RANK decreased instead. OPG and ALP are both osteogenic markers, which indicates bone remodeling status [32]. OPG produced by osteoblasts and osteogenesis progenitor cells can competitively bind to receptor activators of nuclear factor-κB ligand (RANKL) on the surface of osteoclasts, exerting an inhibitory effect on osteoclasts [33]. Moreover, inflammatory cytokines secreted by Th1 and Th17 cells, like IFN-γ and IL-17, amplify the local inflammatory factors production (TNF, IL-1, etc.) and further accelerate the generation of RANKL, promoting osteoclastogenesis (Fig. 8) [34].

The outcome of bone remodeling on tooth extraction in T2DM is closely related to the immune system, as the immune and skeleton systems share a common niche and numerous signaling molecules [35]. Although people believe that the ratio of CD4^+^:CD8^+^T lymphocytes predicts the extent of the immune response, it has been controversial in the alteration of the CD4^+^:CD8^+^T ratio in inflammatory disorders based on previous research [36]. In T2DM, activated CD4^+^ T lymphocytes infiltrate bone marrow and release proinflammatory cytokines, including TNF-α and RANKL, eventually stimulating osteoclast differentiation [4]. Different subsets of T lymphocytes interact with one another synergistically and antagonistically, including Th1, Th2, Th17, and Treg [37]. The function of Th1 and Th2 cells is controlled by transcription factors T-BET and GATA-3, respectively. Th2 cells, characterized as specifically secreting IL-4, IL-5, and IL13, have been considered anti-inflammatory mediators in alveolar bone homeostasis [38]. Th1 cells imply the aggravation of osteoclastogenic through specifically secreting proinflammatory cytokines, including IFN-γ, IL-2, and TNF-α [39].

Moreover, except for its osteogenic properties, ART can also alleviate the incidence of diabetes through increasing the proportion of IL-4-producing CD4^+^T lymphocytes (Th2) while reducing IFN-γ-producing T lymphocytes (Th1) [11]. Our results suggested that the proportion of CD4^+^ and CD8^+^ T lymphocytes and CD4^+^:CD8^+^ T ratio has shown slight changes in both lymph nodes and peripheral blood between the treatment groups and the T2DM group. In addition, compared with the T2DM group, 150 mg/ml ART-loaded TCH has significantly increased the percentages of Th2 while decreasing the percentages of Th1 in cervical lymph nodes. Taken together, ART-loaded TCH promotes osteogenesis and the expression of OPG and ALP via mediating the infiltration of T lymphocyte subsets and inflammatory cytokines.

However, the mechanisms of ART-loaded TCH on tooth extraction sockets of T2DM rats remain unclear. Network pharmacology analysis and molecular docking were conducted to investigate its potential mechanisms. We found that the mitogen-activated protein kinases (MAPKs) signaling pathway plays a vital role in the pathogenesis and ART could build strong interaction bonds with MAPK proteins. The phosphorylation of the MAPK signaling pathway, including ERK, JNK, and p38, initiates downstream inflammatory transcription factor NF-κB, resulting in the activation of immune response and starting a vicious cycle [40]. It can also recruit the infiltration of Th cells and related cytokines (Fig. 8). It has been reported that the phosphorylation of MAPKs is closely associated with osteoclast formation, which can be inhibited by ART [41]. Consistent with previous studies, our results showed that the expression of ERK1 and p38-MAPK and their phosphorylated proteins was highly expressed in T2DM tooth extraction sockets, which was suppressed by 150 mg/ml ART-loaded TCH. Therefore, the MAPK signaling pathway might be the critical mechanism of ART-loaded TCH on osteogenesis in T2DM tooth extraction rats.

However, there are several limitations in the current study. It still needs further to explore the regulation of ART on osteoblasts and osteoclasts. Currently, parts of studies illustrate that ART promotes the osteogenesis of mesenchymal stem cells, but the underlying mechanisms are still unclear [42, 43]. In addition, we have not performed related investigations into the pharmacological properties of ART-loaded TCH, as the joint application of ART and TCH has been well studied [14, 17]. Based on the previous study, our results indicated that 150 mg/ml ART-loaded TCH achieved better therapeutic effects on osteogenesis and anti-inflammation. This may be ascribed to the extent of drug release time. Considering the safety and injection frequency of anesthesia in rats, we have only applied ART-loaded TCH once. It still needs further improvements in the application frequency of medicine and the observation time. To sum up, ART-loaded TCH has promising prospects in promoting osteogenesis and anti-inflammation, which provides an experimental basis for future application in the clinic.

## Conclusion

In conclusion, T2DM tooth extraction rats showed significant bone loss, which can be improved by ART-loaded TCH. Treating 150 mg/ml ART-loaded TCH illustrated greater osteogenesis and anti-inflammation in T2DM rats. In the meanwhile, ART-loaded TCH can increase the percentage of Th2, while decreasing the percentage of Th1. Furthermore, we found that ART-loaded TCH ameliorated bone loss and local T lymphocyte immunoregulation through inhibiting the MAPK signaling pathway.

### Electronic supplementary material

Below is the link to the electronic supplementary material.

Supplementary Material 1

Supplementary Material 2

Supplementary Material 3

## Data Availability

Data is provided within the manuscript and supplementary information files. Supporting information and raw data is available from the first author (Jinghong Luo).

## Abbreviations

IHC: Immunohistochemistry
RT-qPCR: Real-time quantitative polymerase chain reaction
WB: Western Blot
ELISA: Enzyme-Linked Immunosorbent Assays
Th1, Th2, Th17: T helper 1/2/17 cells
Treg: T regulatory cells
CD4: Cluster of differentiation 4
GATA-3: a master regulator of T helper 2 (Th2)-cell differentiation
T-BET: a T-box transcription factor
IFN-γ: Interferon-γ
IL-4: Interleukin-4
OPG: Osteoprotegerin
ALP: Alkaline phosphatase
RIPA: Radio Immunoprecipitation Assay
SDS-PAGE: Sodium dodecyl sulfate-polyacrylamide gel electrophoresis
PVDF: Polyvinylidene difluoride
HRP: Horseradish peroxidase
ECL: Chemiluminescence substrate
