# Supplementary material for: Artesunate-loaded thermosensitive chitosan hydrogel promotes osteogenesis of maxillary tooth extraction through regulating T lymphocytes in type 2 diabetic rats
Source: BMC Oral Health. 2024 Mar 20;24:356. doi: 10.1186/s12903-024-04127-7 (PMC10953264; doi:10.1186/s12903-024-04127-7)
Supplement: Supplementary file 1 — Supplementary Material 1 [file 12903_2024_4127_MOESM1_ESM.docx]

**Additional file 1 Antibody Composition of Two Panels**

| Fluorochrome | eFluor^TM^450 | PerCP-eFluor^TM^710 | FITC | PE-Cyanine7 | PE |
| --- | --- | --- | --- | --- | --- |
| Panel 1 | Dead/Live dye | CD3 | CD4 | CD8 |  |
| Panel 2 | Dead/Live dye | CD3 | CD4 | CD8 | IL-4 |

IL-4 (BD Pharmingen, No.555082)

CD3 (eBioscience, Invitrogen, Catalog No. 46-0030-82)

CD4 (eBioscience, Invitrogen, Catalog No. 11-0040-82)

CD8a (eBioscience, Invitrogen, Catalog No. 25-0084-82)

IgG2 kappa Isotype Control PE(eBioscience, Invitrogen, Catalog No. 12-4321-80)

Cell stimulation cocktail (eBioscience, Invitrogen, Catalog No.00-4975)

Fixable viability dye eFluor^TM^450 (eBioscience, Invitrogen, Catalog No.65-0863)
