# Supplementary material for: Artesunate-loaded thermosensitive chitosan hydrogel promotes osteogenesis of maxillary tooth extraction through regulating T lymphocytes in type 2 diabetic rats
Source: BMC Oral Health. 2024 Mar 20;24:356. doi: 10.1186/s12903-024-04127-7 (PMC10953264; doi:10.1186/s12903-024-04127-7)
Supplement: Supplementary file 2 — Supplementary Material 2 [file 12903_2024_4127_MOESM2_ESM.docx]

| **Additional file 2 Primer used for RT-qPCR** | | |
| --- | --- | --- |
| Gene | Forward (5’ to 3’) | Reverse (5’ to 3’) |
| GATA-3 | GATGCTTGCTGTTCTCGGGT | GTCCCAGTCACCCCTCCATT |
| IL-4 | TCCACGGATGTAACGACAGC | TGGTGTTCCTTGTTGCCGTA |
| T-BET | TTGGAAGGTGCCCGACTAAC | GAAGTCCCTCTGGGTCCTAA |
| IL-10 | AATCTGTGTTGTTTAAGCTGTTTCC | TTTATTCAAAACGAGGATCTGCTAC |
| IFN-γ | GCCAAGTTCGAGGTGAACAAC | CAGAATCAGCACCGACTCCTT |
| TNF-α | ATACACTGGCCCGAGGCAAC | GTTTGCTACGACGTGGGCTA |
| ERK | GGACACAAGGACTGCCACTG | GGTGGTAAGCAGCCAGTTGT |
| MAPK | GGCCGCGCTACACTAATCTC | TCTCATGTCTGAAGCGCAGT |
| ALP | TGGCAGTGGTATTGTAGGTGC | CTCCCCCTCCACGAAGAAGT |
| OPG | CGTCATCGAAAGCACCCTGT | TGGTAGGCACAGCAAACCTG |
| β-actin | AGAGGGAAATCGTGCGTGACA | CGATAGTGATGACCTGACCGTCA |
