# Supplementary material for: Artesunate-loaded thermosensitive chitosan hydrogel promotes osteogenesis of maxillary tooth extraction through regulating T lymphocytes in type 2 diabetic rats
Source: BMC Oral Health. 2024 Mar 20;24:356. doi: 10.1186/s12903-024-04127-7 (PMC10953264; doi:10.1186/s12903-024-04127-7)

Additional file 3 The protein expression of GATA-3, IL-4, T-BET, and IFN-γ in cervical lymph nodes and the protein expression of p38-MAPK, p-p38-MAPK, ERK1, and p-ERK1 in maxilla were detected by WB. The uncropped blots used in the figures are displayed.

GATA-3 (50kDa)

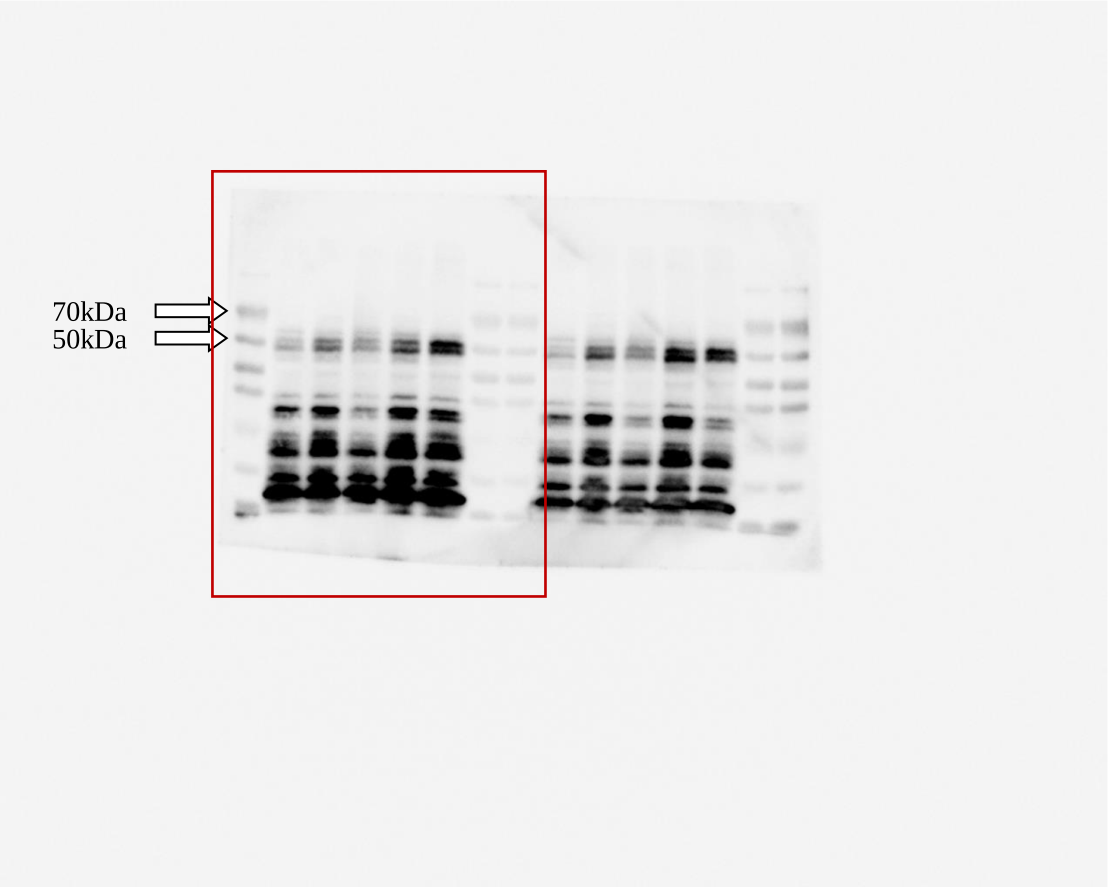

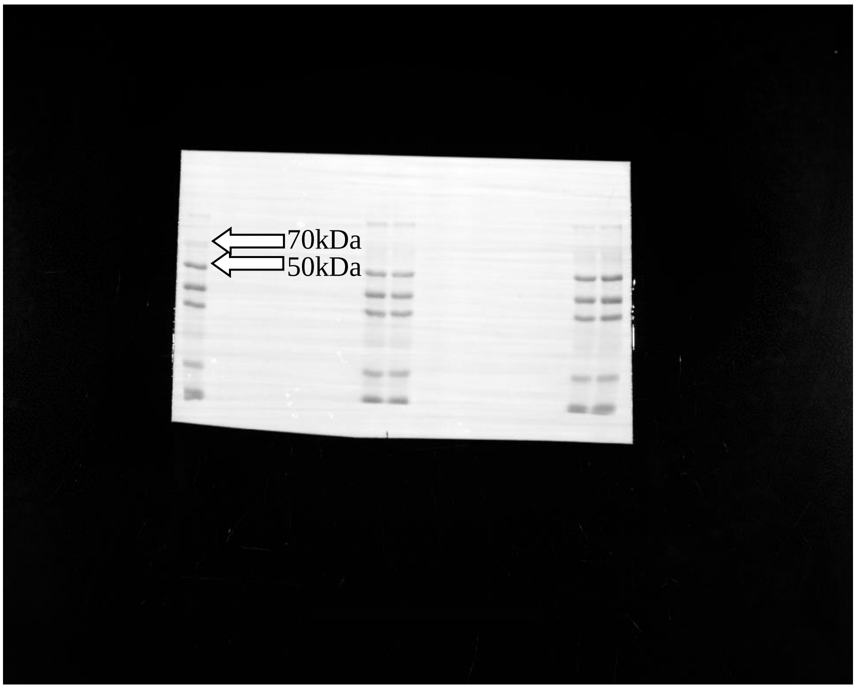

β-actin (43kDa) and IL-4 (20kDa)

The blot on the upside is incubated with β-actin, and the blot on the downside is incubated with IL-4.

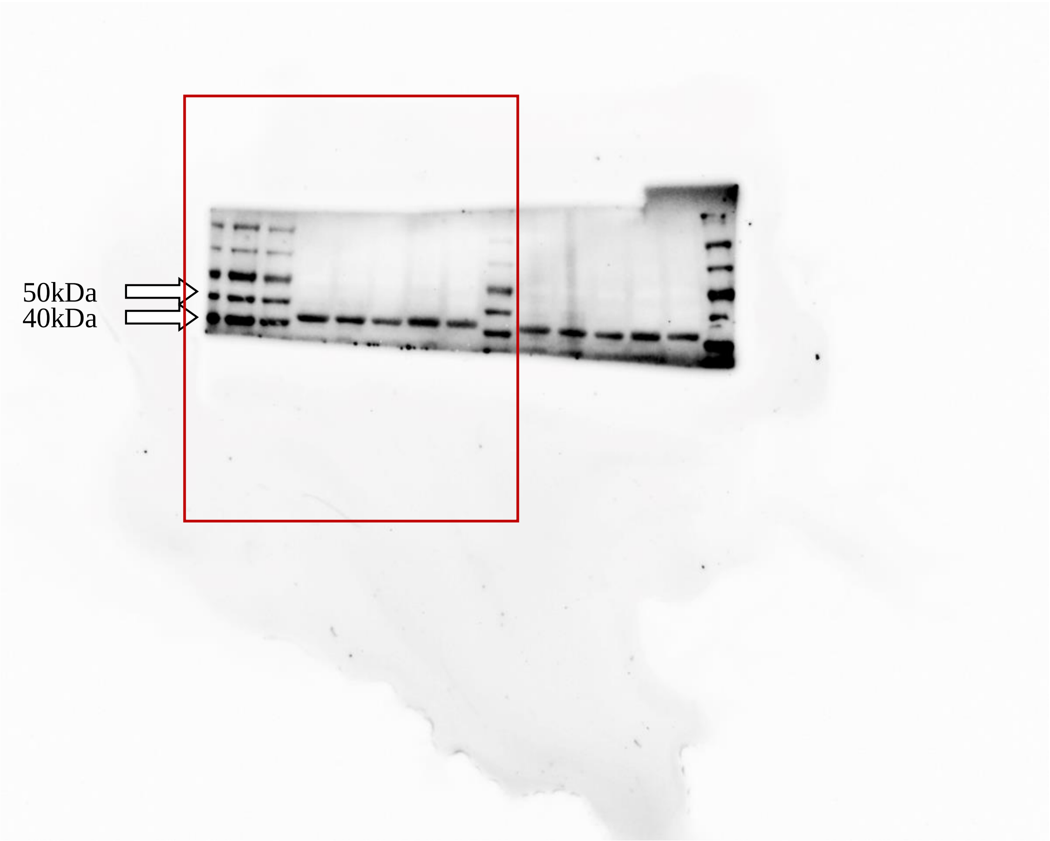

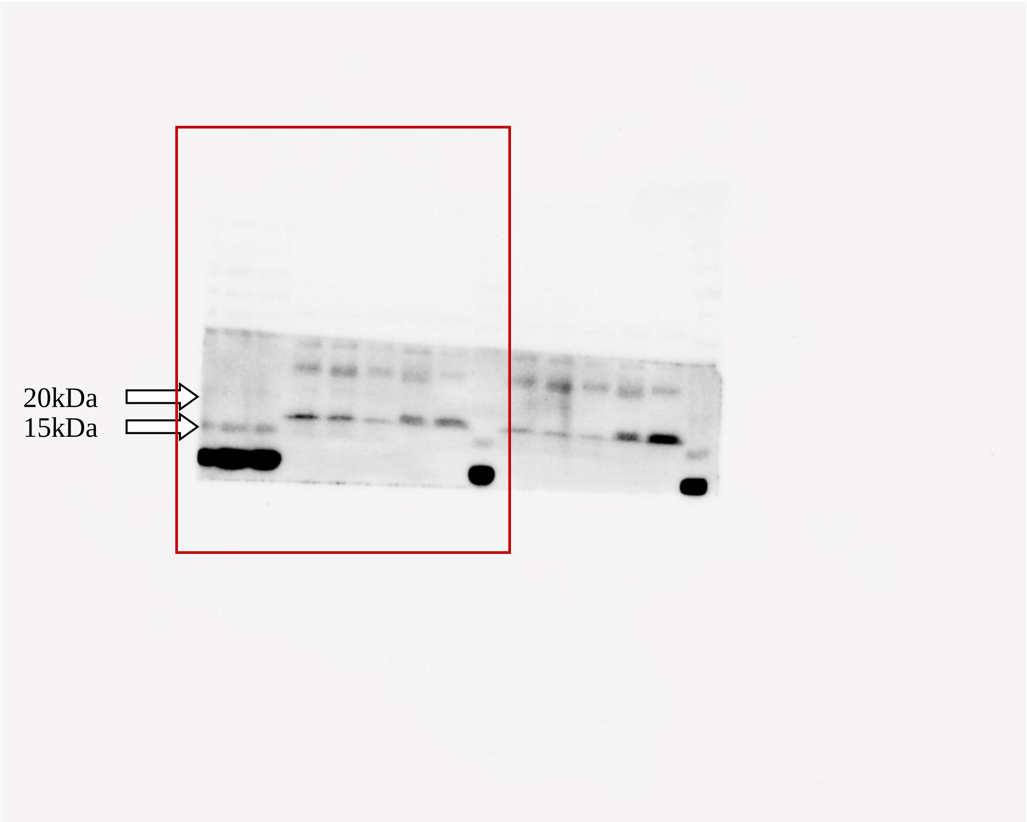

T-BET (58kDa)

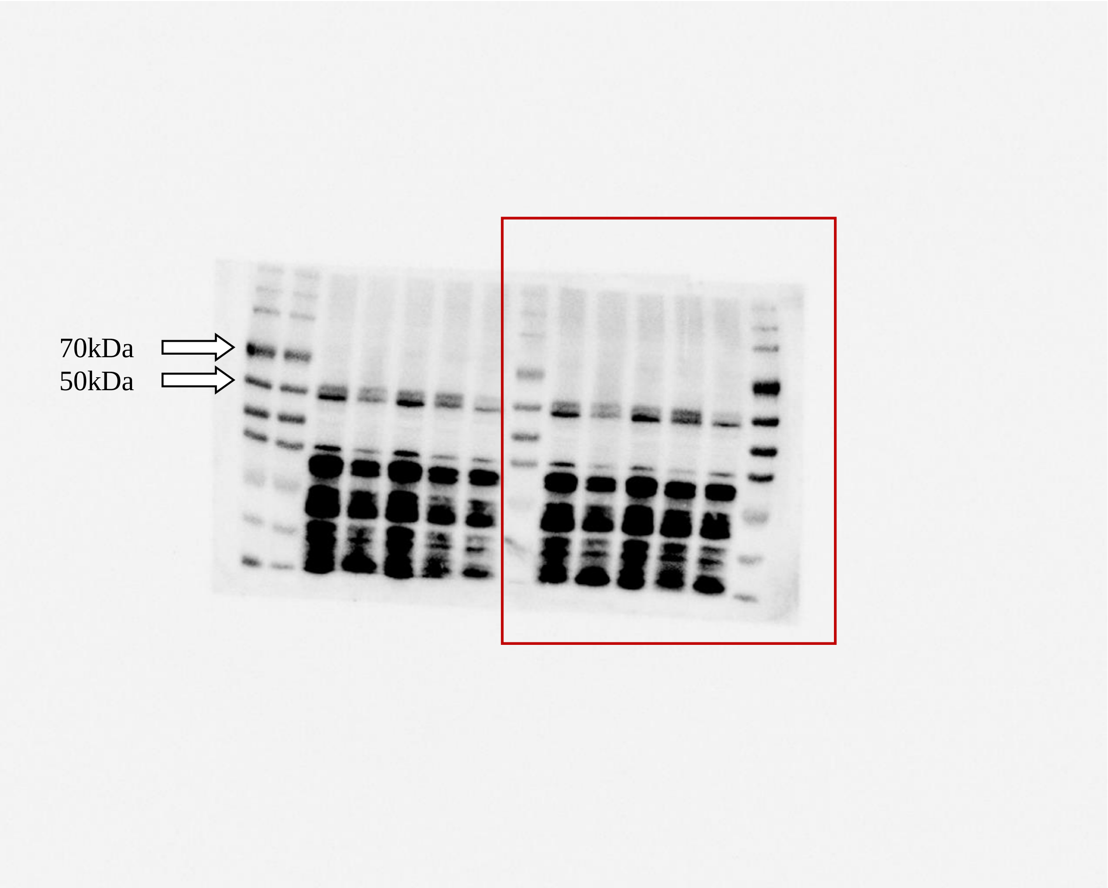

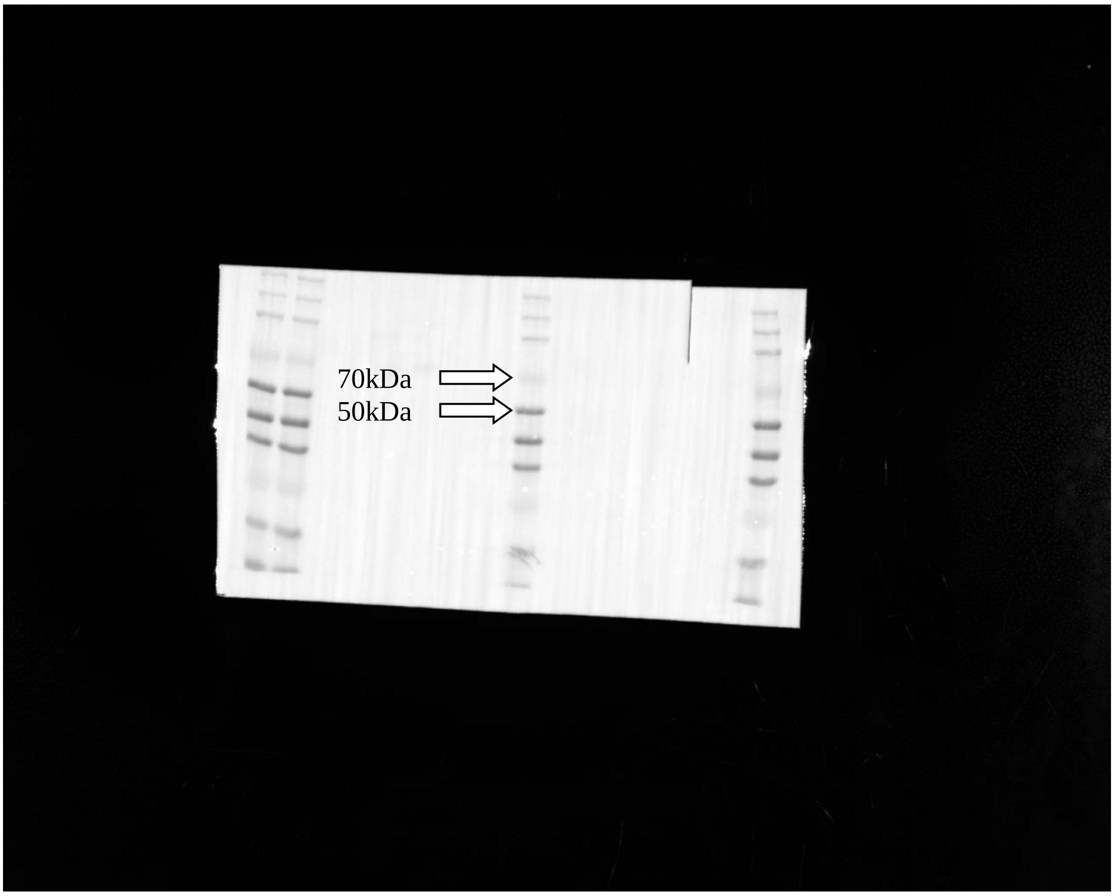

p38 MAPK (42kDa)

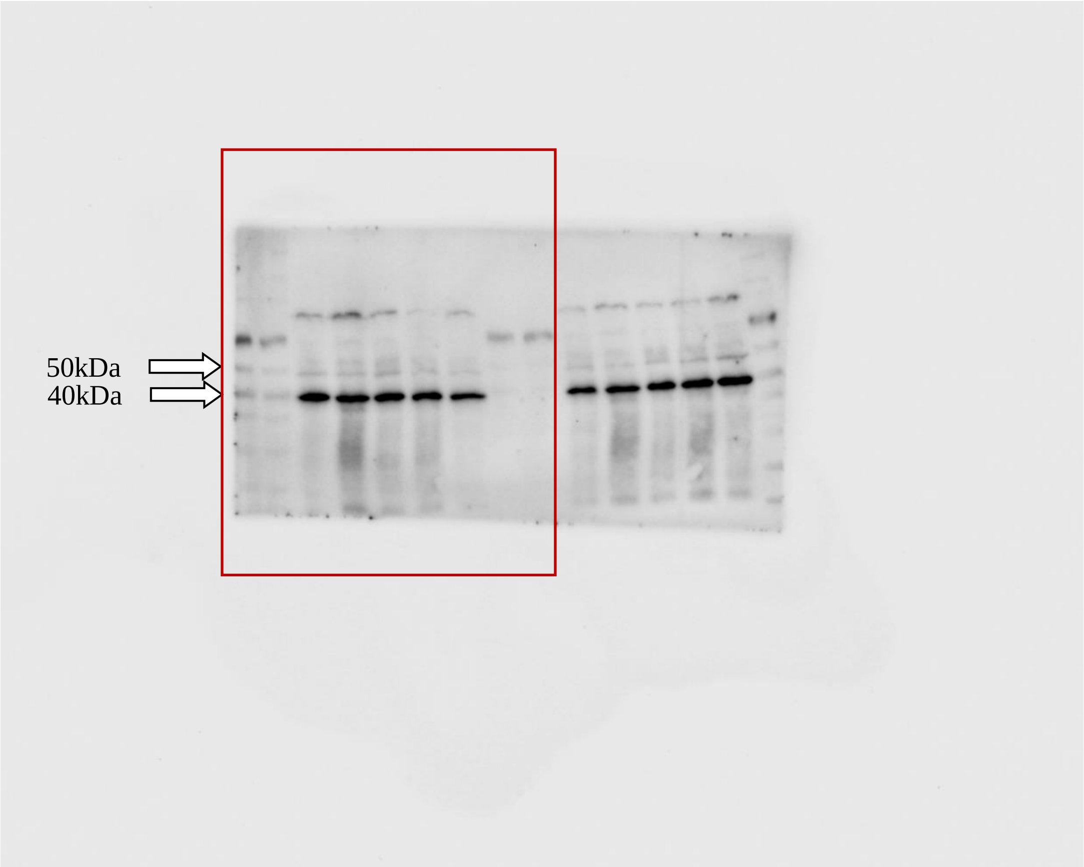

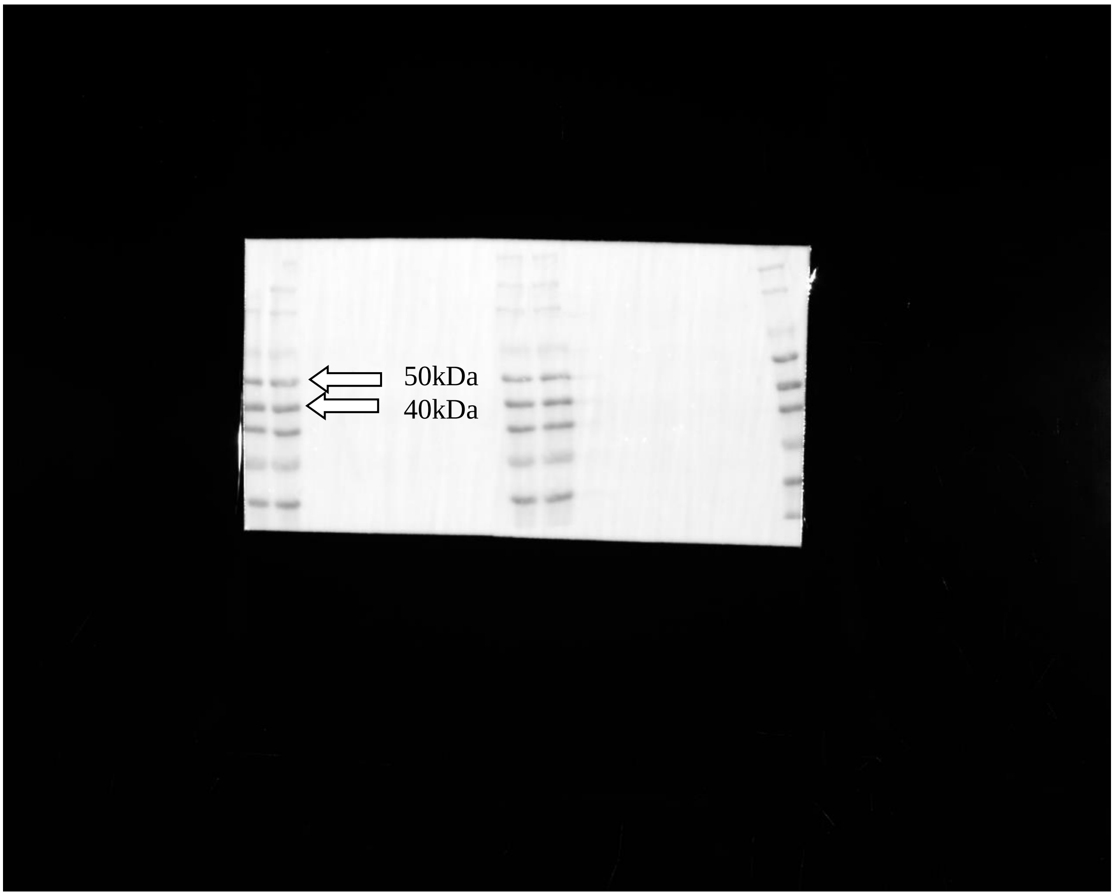

p-p38 (42kDa)

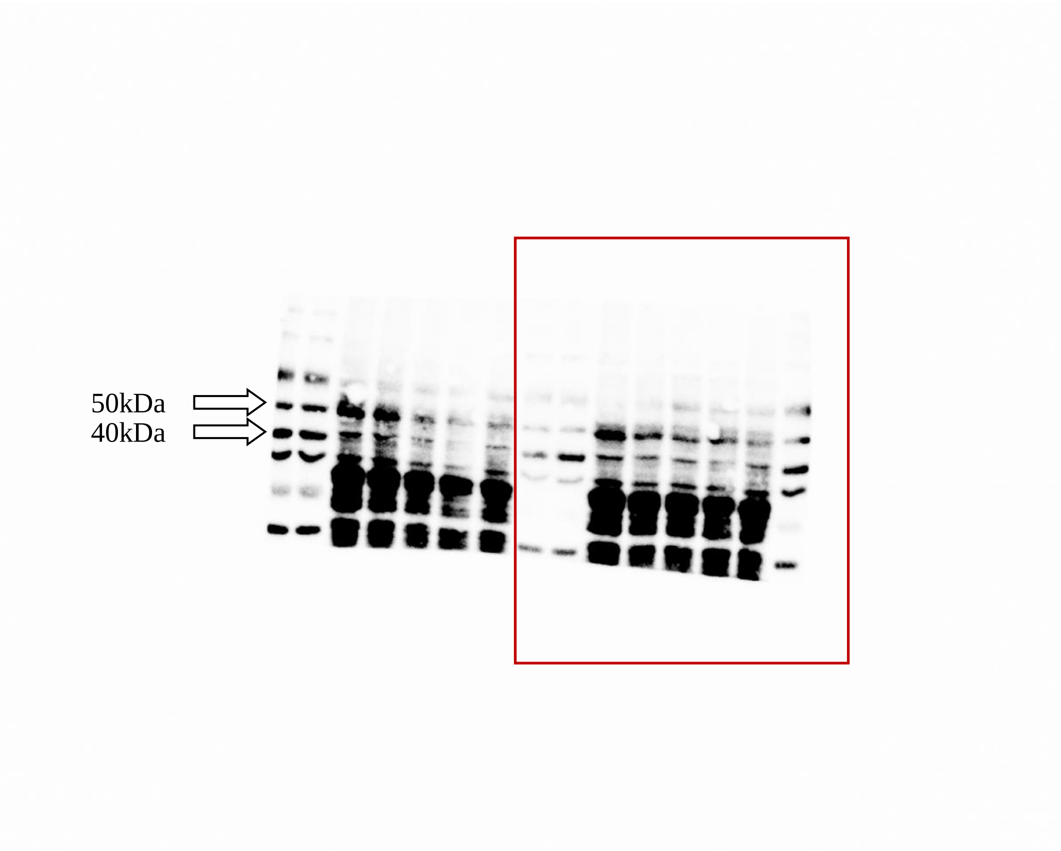

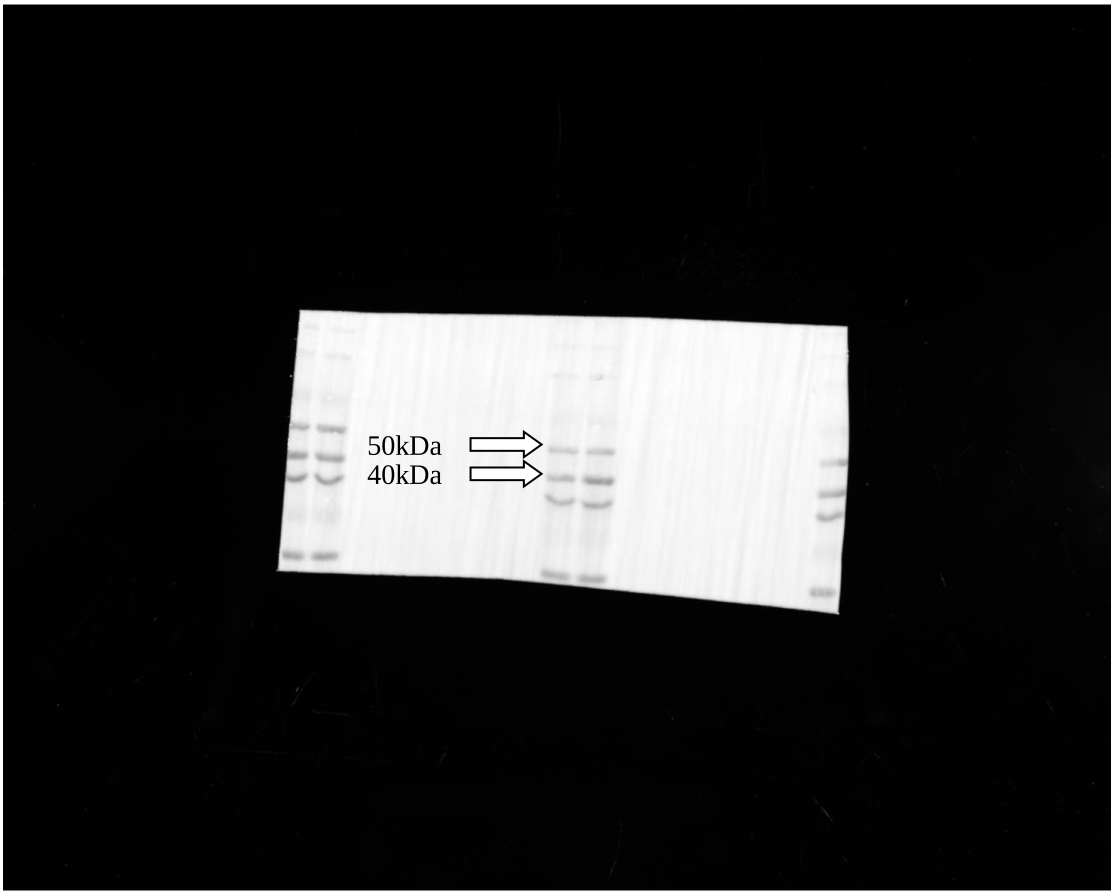

β-actin (42kDa)

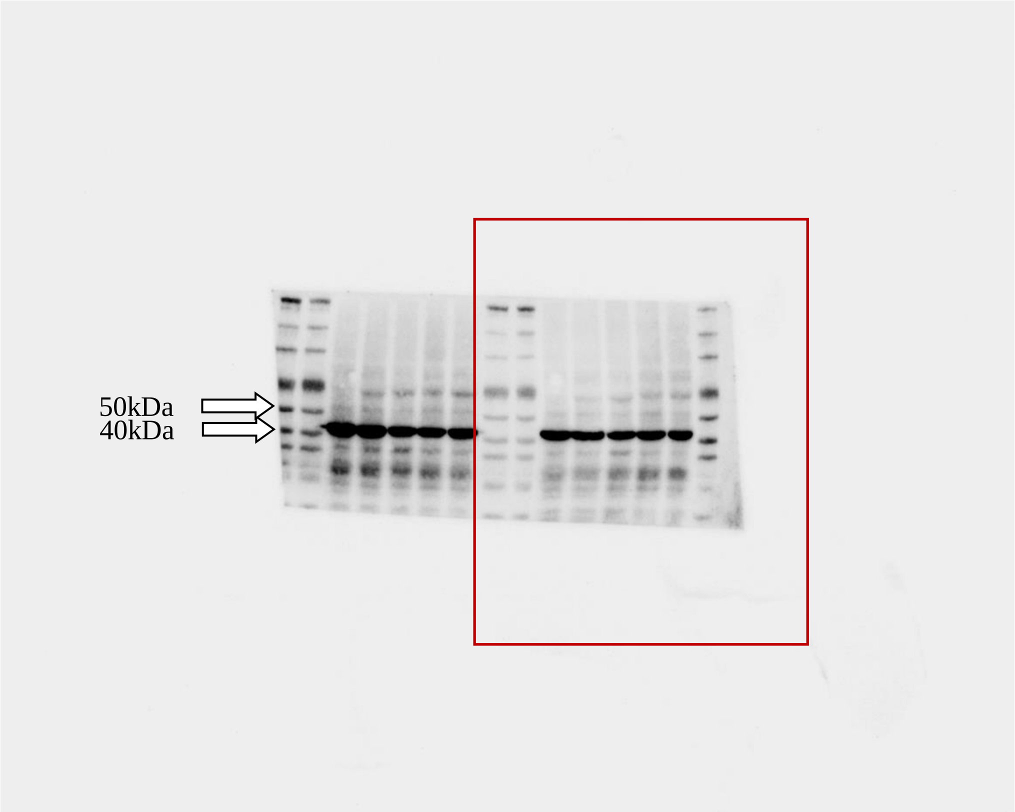

ERK1 (43kDa)

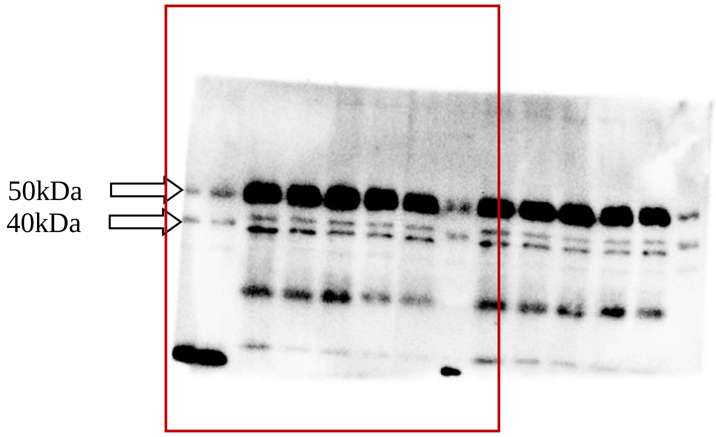

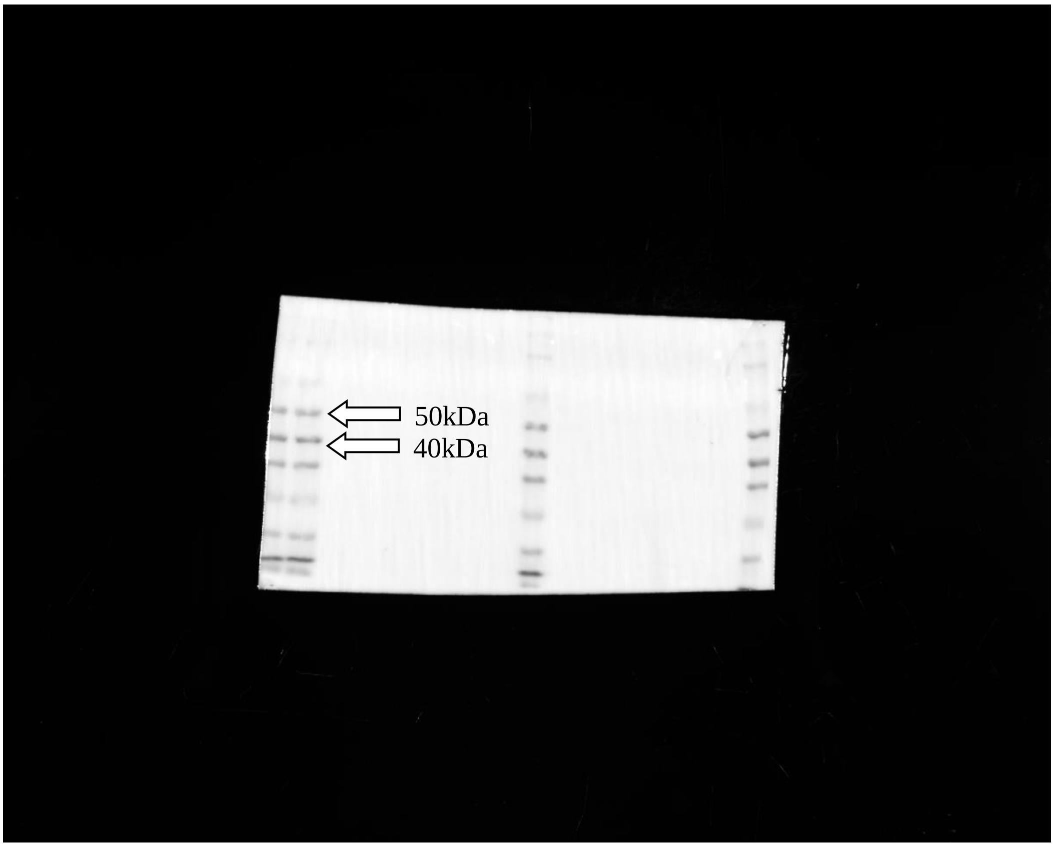

p-ERK1 (43kDa)

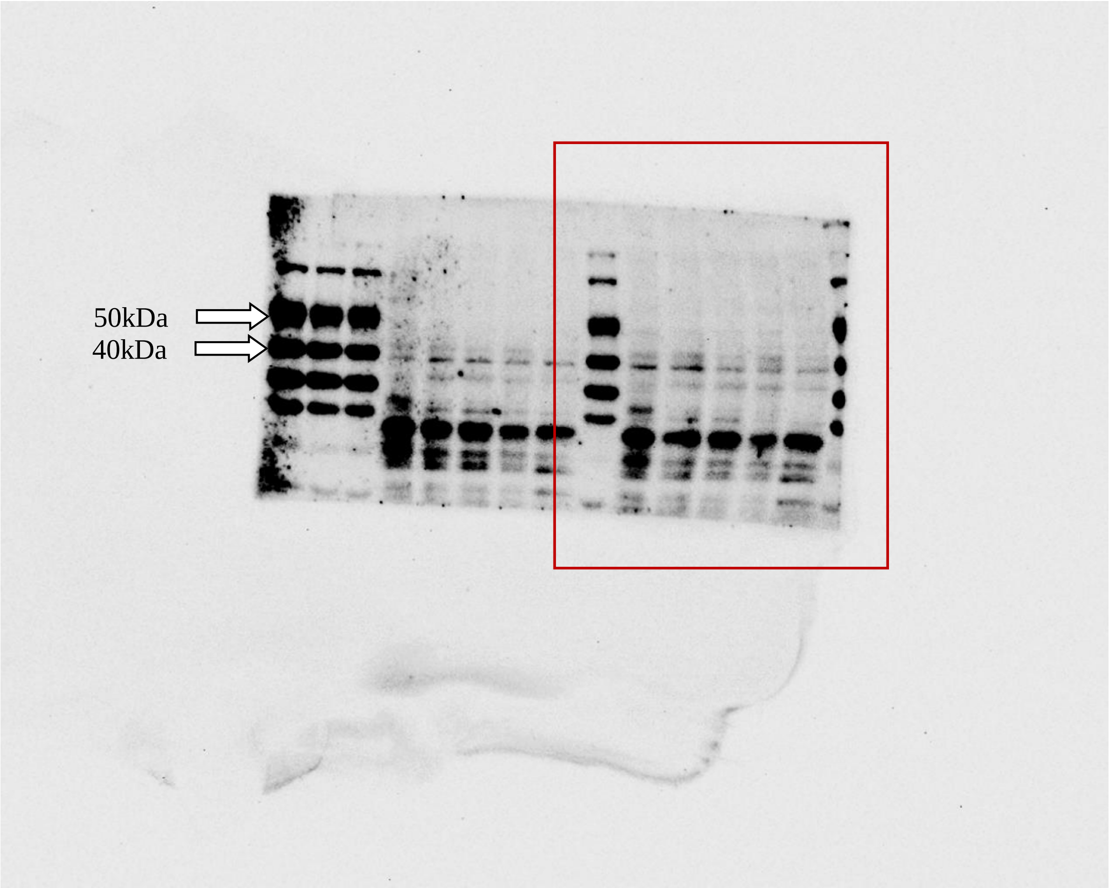

β-actin (42kDa)

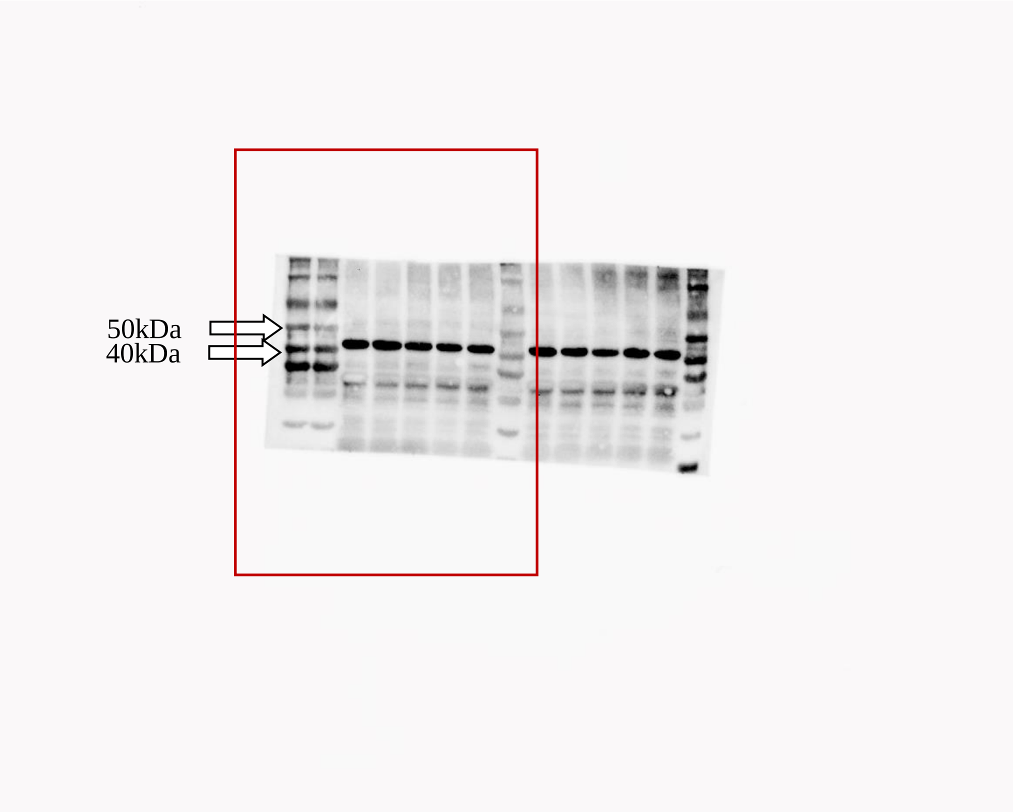

Supplement: Supplementary file 3 — Supplementary Material 3 [file 12903_2024_4127_MOESM3_ESM.pdf]
